# Synthesis, central nervous system activity, and structure–activity relationship of 1-aryl-6-benzyl-7-hydroxy-2,3-dihydroimidazo[1,2-a]pyrimidine-5(1*H*)-ones

**DOI:** 10.1007/s00044-014-0993-1

**Published:** 2014-03-27

**Authors:** Marzena Rządkowska, Elżbieta Szacoń, Agnieszka A. Kaczor, Sylwia Fidecka, Ewa Kędzierska, Dariusz Matosiuk

**Affiliations:** 1 Department of Synthesis and Chemical Technology of Pharmaceutical Substances with Computer Modeling Lab, Faculty of Pharmacy with Division of Medical Analytics, Medical University of Lublin, 4A Chodźki St, 20093 Lublin, Poland; 2 School of Pharmacy, University of Eastern Finland, Yliopistonranta 1, P.O. Box 1627, 70211 Kuopio, Finland; 3 Department of Pharmacology and Pharmacodynamics, Faculty of Pharmacy with Division of Medical Analytics, Medical University of Lublin, 4A Chodźki St, 20093 Lublin, Poland

**Keywords:** Antinociceptive compounds, Central nervous system activity, Imidazo[1,2-a]pyrimidines

## Abstract

A series of 24 1-aryl-6-benzyl-7-hydroxy-2,3-dihydroimidazo[1,2-a]pyrimidine-5(1*H*)-ones was designed as antinociceptive compounds acting through opioid receptors with additional serotoninergic activity. The compounds, similarly as previously published series, lack the protonable nitrogen atom which is a part of classical opioid receptor pharmacophore and is necessary to interact with the conserved Asp(3.32) in the opioid receptor binding pocket. The compounds were obtained in one-step cyclocondensation of 1-aryl-4,5-dihydro-1*H*-imidazol-2-amines diethyl 2-benzylmalonate or diethyl 2-(2-chlorobenzyl)malonate under basic conditions. Almost all the tested compounds exerted strong antinociceptive activity, but surprisingly, it was not reversed by naloxone; thus, it is not mediated through opioid receptors. It makes it possible to conclude that addition of one more aromatic moiety to the non-classical opioid receptor pharmacophore results in the compounds which are not opioid receptor ligands. The lack of activity of one of the tested compounds may be attributed to low blood–brain barrier permeation or unfavorable distribution of electrostatic potential and HOMO and LUMO orbitals.

**Electronic supplementary material:**

The online version of this article (doi:10.1007/s00044-014-0993-1) contains supplementary material, which is available to authorized users.

## Introduction

**Fig. 1:**
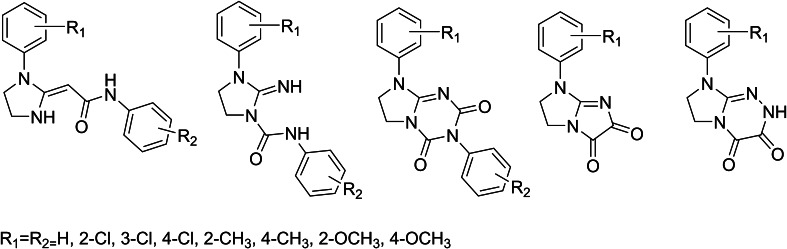
Antinociceptive compounds following the non-classical opioid receptor pharmacophore models. All the series have been reported with the given set of substituents

**Fig. 2:**
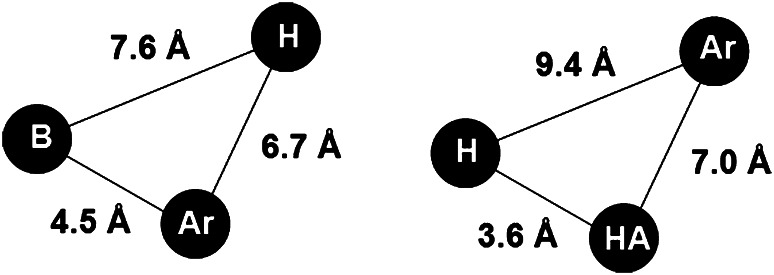
The non-classical opioid receptor models. *B* base, *H* hydrophobic group, *Ar* aromatic group, *HA* hydrogen bond acceptor

**Fig. 3:**
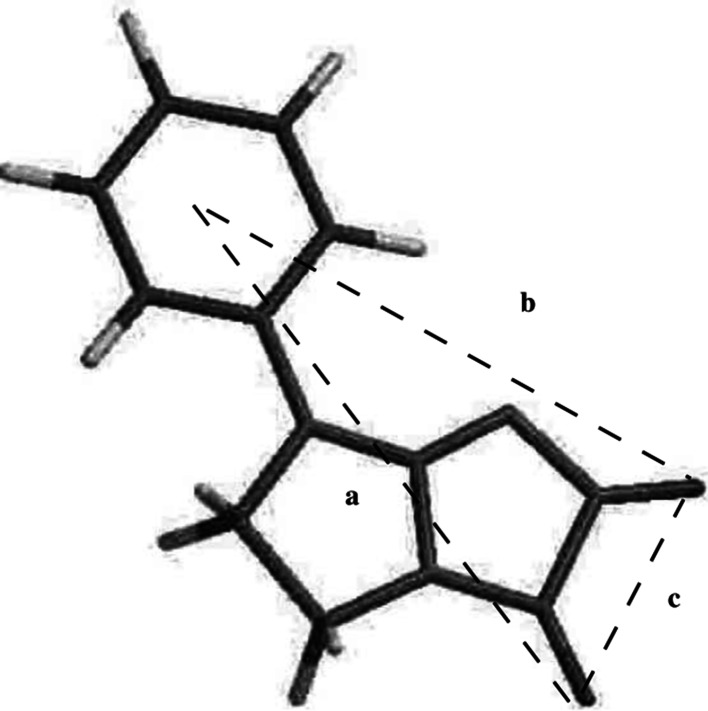
The pharmacophore model for the affinity to 5-HT_2_ receptor (Matosiuk *et al.*, 2002b) consisting of an aromatic moiety and two hydrogen bond acceptors

The treatment of central nervous system diseases in European Union costs 386 billion euro per year, placing these diseases among the most costly medical conditions (Di Luca *et al.*, 2011). In particular, treatment of pain is an extremely important medical problem with social and economic implications. Searching for new antinociceptive agents follows nowadays two main strategies: exploitation of well-established targets, such as opioid receptors (Kaczor and Matosiuk, 2002a, b) or identification of novel molecular targets. In our continuous efforts to find novel antinociceptive agents, we synthesized and studied several series of novel heterocyclic compounds acting through opioid receptors, Fig. 1 (Matosiuk *et al.*, 2001, 2002a, b; Sztanke *et al.*, 2005). Many morphine-like narcotic analgesics share in their structure similar features, which are the phenyl ring, tertiary nitrogen atom, and the two carbon fragment (e.g., as a part of the piperidine ring). This classical opioid pharmacophore model was one of the first models used to explain the antinociceptive activity of morphine derivatives. Interestingly, the compounds presented in Fig. 1, similarly as salvinorin A (a potent κ opioid receptor ligand) do not possess a protonable nitrogen atom, capable to interact with the conserved aspartate residue (Asp3.32) in the receptor binding pocket. Instead, these compounds follow the non-classical opioid receptor pharmacophore models as presented in Fig. 2, which involve a base (B), a hydrophobic (H) and aromatic moiety (Ar) or hydrogen bond acceptor (HA), hydrophobic (H), and aromatic groups (Ar) (Huang *et al.,* 1997; Matosiuk *et al.*, 2001, 2002a, 2002b; Sztanke *et al.*, 2005). In addition to the antinociceptive activity, some of the compounds presented in Fig. 1 exhibited also serotoninergic activity and affinity to 5-HT_2_ serotonin receptor. It was proposed that two hydrogen bond donors and the aromatic moiety are required for the serotoninergic activity as presented in Fig. 3 (Matosiuk *et al.*, 2002b).

Based on our previous results, we designed a series of 1-aryl-6-benzyl-7-hydroxy-2,3-dihydroimidazo[1,2-a]pyrimidine-5(1*H*)-ones (Rządkowska *et al.*, 2009). The rationale of the study may be summarized as follows: (a) the designed compounds fulfilled both non-classical opioid receptor pharmacophore models presented in Fig. 2 as well as the model for serotoninergic activity depicted in Fig. 3; (b) the designed series is aimed to determine the effect of the second aromatic moiety on the antinociceptive activity; (c) the designed compounds were expected to have favorable values of lipohilicity and ADMET parameters for the activity in central nervous system; (d) the imidazo[1,2-a]pyrimidine scaffold is present in many biologically active compounds which have been reported to exhibit not only central nervous system activity (Blackaby *et al.*, 2006; Goodacre *et al.*, 2006; Jensen *et al.,* 2005; Matosiuk, *et al.*, 1996; Tully *et al.*, 1991) but also anti-inflammatory and analgesic (Abignente *et al.*, 1994; Freeman *et al.,* 1978; Sacchi *et al.*, 1997; Vidal *et al.,* 2001), antibacterial (Al-Tel and Al-Qawasmeh, 2010; Moraski *et al.,* 2012; Rival *et al.*, 1992; Steenackers *et al.*, 2011a, b), antiviral (Gueiffier *et al.*, 1996), antifungal (Rival *et al.*, 1991, 1993), insecticidal, acaricidal and nematocidal (Dehuri *et al.*, 1983), hormonal (Sasaki *et al.*, 2002), mutagenic (Turner *et al.*, 1978), anticancer (Guo *et al.*, 2011; Lin *et al.*, 2012; Linton *et al.,* 2011), and cardiovascular (Okabe *et al.*, 1983) activity; (e) the set of substituents was similar to those in previously reported series (Fig. 1) which turned out to exhibit the expected profile of pharmacological activity.

In this study, we present synthesis, computational drug-likeness estimation and ADMET pre-screening, pharmacological activity determination, and some structure–activity relationship studies for the series of 24 1-aryl-6-benzyl-7-hydroxy-2,3-dihydroimidazo[1,2-a]pyrimidine-5(1*H*)-ones. The main finding of the studies is that although all the investigated compounds exhibited strong antinociceptive properties, this activity was not reversed by naloxone; thus, it is not mediated through opioid receptors.

## Materials and methods

### Chemistry

Reactions were routinely monitored by thin-layer chromatography (TLC) in silica gel (60 F_254_ Merck plates), and the products were visualized with ultraviolet light of 254 nm wavelength. All NMR spectra were acquired on Bruker Fourier 300 MHz spectrometer. Spectra were recorded at 25 °C using DMSO as a solvent with a non-spinning sample in 5 mm NMR-tubes. MS spectra were recorded on Bruker microTOF-Q II and processed using Compass Data Analysis software. The elementary analysis was performed with the application of Perkin-Elmer analyzer. Melting points were determined with Boetius apparatus.

### General procedure to obtain compounds **3a**–**3x**

0.02 mol of hydrobromide of 1-aryl-4,5-dihydro-1*H*-imidazol-2-amines (**1a**–**1l**), 0.02 mol of diethyl 2-benzylmalonate (**2a**), or diethyl 2-(2-chlorobenzyl)malonate (**2b**), 15 mL of 16.7 % solution of sodium methoxide, and 60 mL of methanol were heated in a round-bottom flask equipped with a condenser and mechanic mixer in boiling for 8 h. The reaction mixture was then cooled down, and the solvent was distilled off. The resulted solid was dissolved in 100 mL of water, and 10 % solution of hydrochloric acid was added till acidic reaction. The obtained precipitation was filtered out, washed with water, and purified by crystallization from methanol.

#### 6-Benzyl-1-phenyl-7-hydroxy-2,3-dihydroimidazo[1,2-a]pyrimidine-5(1*H*)-one (**3a**)

0.02 mol (4.84 g) of hydrobromide of 1-phenyl-4,5-dihydro-1*H*-imidazol-2-amine (**1a**), 0.02 mol (5.0 g) of diethyl 2-benzylmalonate (**2a**), 15 mL of 16.7 % solution of sodium methoxide and 60 mL of methanol were heated in a round-bottom flask equipped with a condenser and mechanic mixer in boiling for 8 h. The reaction mixture was then cooled down, and the solvent was distilled off. The resulted solid was dissolved in 100 mL of water, and 10 % solution of hydrochloric acid was added till acidic reaction. The obtained precipitation was filtered out, washed with water, and purified by crystallization from methanol. It was obtained 2.81 g of **3a** (44 % yield), white crystalline solid, m.p. 278–280 °C; ^1^H NMR (DMSO-*d*_6_, 300 MHz,): *δ* = 10.90 (s, 1H, OH), 7.05–7.88 (m, 10H, CH_arom_.), 4.11 (dd, 2H, *J* = 9.0, *J*′ = 7.6 Hz, H_2_-2), 4.17 (dd, 2H, *J* = 9.0, *J*′ = 7.6 Hz, H_2_-2), 3.63 (s, 2H, CH_2benzyl_); ^13^C NMR (DMSO-*d*_6_, 75 MHz,): *δ* = 26.1 (CBz), 40.4 (C-2), 43.2 (C-3), 91.6 (C-6), 111.4, 112.2, 112.5, 122.1, 127.3, 127.8, 128.4, 128.7, 152.4 (C-7), 164.6 (C-8a), 168.5 (C-5),; EIMS *m/z* 320.1 [M+H]^+^. HREIMS (*m/z*): 319.1049 [M^+^] (calcd. for C_19_H_17_N_3_O_2_ 319.3690); Anal. calcd. for: C_19_H_17_N_3_O_2_ C, 71.45; H, 5.36; N, 13.16. Found C, 70.96; H, 5.88; N, 13.14.

#### 6-Benzyl-1-(2-chlorphenyl)-7-hydroxy-2,3-dihydroimidazo[1,2-a]pyrimidine-5(1*H*)-one (**3b**)

0.02 (5.49 g) mol of hydrobromide of 1-(2-chlorphenyl)-4,5-dihydro-1*H*-imidazol-2-amine (**1b**), 0.02 mol (5.0 g) of diethyl 2-benzylmalonate (**2a**), 15 mL of 16.7 % solution of sodium methoxide and 60 mL of methanol were heated in a round-bottom flask equipped with a condenser and mechanic mixer in boiling for 8 h. The reaction mixture was then cooled down, and the solvent was distilled off. The resulted solid was dissolved in 100 mL of water, and 10 % solution of hydrochloric acid was added till acidic reaction. The obtained precipitation was filtered out, washed with water, and purified by crystallization from methanol. It was obtained 5.94 g of **3b** (84 % yield), white crystalline solid, m.p. 283–285 °C; ^1^H NMR (DMSO-*d*_6_, 300 MHz,): *δ* = 11.04 (s, 1H, OH), 7.10–8.06 (m, 9H, CH_arom_.), 4.06 (dd, 2H, *J* = 8.9, *J*′ = 7.5 Hz, H_2_-2), 4.22 (dd, 2H, *J* = 8.9, *J*′ = 7.5 Hz,H_2_-2), 3.60 (s, 2H, CH_2benzyl_); ^13^C NMR (75 MHz, DMSO-*d*_6_): *δ* = 28.5 (CBz), 40.3 (C-2), 45.3 (C-3), 93.6 (C-6), 117.2, 118.5, 123.1, 125.8, 128.4, 128.7, 130.8, 130.8, 141.2, 142.3, 151.4 (C-7), 162.6 (C-8a), 166.6 (C-5),; EIMS *m/z* 354.1 [M+H]^+^. HREIMS (*m/z*): 353.1046 [M^+^] (calcd. for C_19_H_16_ClN_3_O_2_ 353.8180); Anal. calcd. for: C_19_H_16_ClN_3_O_2_C, 64.50; H, 4.56; Cl, 10.02; N, 11.88. Found C, 63.89; H, 4.49;Cl, 10.18; N, 11.80.

#### 6-Benzyl-1-(3-chlorphenyl)-7-hydroxy-2,3-dihydroimidazo[1,2-a]pyrimidine-5(1*H*)-one (**3c**)

0.02 mol (5.49 g) of hydrobromide of 1-(3-chlorphenyl)-4,5-dihydro-1*H*-imidazol-2-amine (**1c**), 0.02 mol (5.0 g) of diethyl 2-benzylmalonate (**2a**), 15 mL of 16.7 % solution of sodium methoxide and 60 mL of methanol were heated in a round-bottom flask equipped with a condenser and mechanic mixer in boiling for 8 h. The reaction mixture was then cooled down, and the solvent was distilled off. The resulted solid was dissolved in 100 mL of water, and 10 % solution of hydrochloric acid was added till acidic reaction. The obtained precipitation was filtered out, washed with water, and purified by crystallization from methanol. It was obtained 6.22 g of **3c** (88 % yield), white crystalline solid, m.p. 278–280 °C; ^1^H NMR (DMSO-*d*_6_, 300 MHz,): *δ* = 10.94 (s, 1H, OH), 7.15–7.85 (m, 9H, CH_arom_), 4.00 (dd, 2H, *J* = 9.0, *J*′ = 7.4 Hz, H_2_-2), 4.16 (dd, 2H, *J* = 9.0, *J*′ = 7.4 Hz, H_2_-2), 3.36 (s, 2H, CH_2benzyl_);^13^C NMR (DMSO-*d*_6_, 75 MHz,): *δ* = 26.1 (CBz), 40.8 (C-2), 42.6 (C-3), 93.3 (C-6), 118.2, 118.5, 121.5, 124.6, 126.4, 126.7, 129.0, 131.3, 131.8, 152.3 (C-7), 162.3 (C-8a), 166.8 (C-5),; EIMS *m/z* 354.1 [M+H]^+^. HREIMS (*m/z*): 353.1064 [M^+^] (calcd. for C_19_H_16_ClN_3_O_2_ 353.8180); Anal. calcd. for C_19_H_16_ClN_3_O_2_ C, 64.50; H, 4.56; Cl, 10.02; N, 11.88. Found C, 64.33; H, 4.52; Cl, 10.02; N, 11.90.

#### 6-Benzyl-1-(4-chlorphenyl)-7-hydroxy-2,3-dihydroimidazo[1,2-a]pyrimidine-5(1*H*)-one (**3d**)

0.02 mol (5.49 g) of hydrobromide of 1-(4-chlorphenyl)-4,5-dihydro-1*H*-imidazol-2-amine (**1d**), 0.02 mol (5.0 g) of diethyl 2-benzylmalonate (**2a**), 15 mL of 16.7 % solution of sodium methoxide and 60 mL of methanol were heated in a round-bottom flask equipped with a condenser and mechanic mixer in boiling for 8 h. The reaction mixture was then cooled down, and the solvent was distilled off. The resulted solid was dissolved in 100 mL of water, and 10 % solution of hydrochloric acid was added till acidic reaction. The obtained precipitation was filtered out, washed with water, and purified by crystallization from methanol. It was obtained 3.95 g of **3d** (56 % yield), white crystalline solid, m.p. 295–297 °C; ^1^H NMR (DMSO-*d*_6_, 300 MHz,): *δ* = 11.05 (s, 1H, OH), 7.09–7.89 (m, 9H, CH_arom_), 4.07 (dd, 2H, *J* = 9.1, *J*′ = 7.6 Hz, H_2_-2), 4.22 (dd, 2H, *J* = 9.1, *J*′ = 7.6 Hz, H_2_-2), 3.58 (s, 2H, CH_2benzyl_); ^13^C NMR (DMSO-*d*_6_, 75 MHz,): *δ* = 24.2 (CBz), 40.4 (C-2), 42.5 (C-3), 93.9 (C-6), 117.3, 118.0, 119.1, 121.2, 124.8, 125.4, 126.9, 129.2, 130.2, 130.7, 151.9 (C-7), 162.4 (C-8a), 166.9 (C-5),; EIMS *m/z* 354. [M+H]^+^. HREIMS (*m/z*): 353.1061 [M^+^] (calcd. for C_19_H_16_ClN_3_O_2_ 353.8180); Anal. calcd. for C_19_H_16_ClN_3_O_2_: C, 64.50; H, 4.56; Cl, 10.02; N, 11.88. Found C, 64.23 %; H, 4.67; Cl, 10.01; N, 11.80.

#### 6-Benzyl-1-(3,4-dichlorphenyl)-7-hydroxy-2,3-dihydroimidazo[1,2-a]pyrimidine-5(1*H*)-one (**3e**)

0.02 (6.18 g) mol of hydrobromide of 1-(3,4-dichlorphenyl)-4,5-dihydro-1*H*-imidazol-2-amine (**1e**), 0.02 mol (5.0 g) of diethyl 2-benzylmalonate (**2a**), 15 mL of 16.7 % solution of sodium methoxide and 60 mL of methanol were heated in a round-bottom flask equipped with a condenser and mechanic mixer in boiling for 8 h. The reaction mixture was then cooled down, and the solvent was distilled off. The resulted solid was dissolved in 100 mL of water, and 10 % solution of hydrochloric acid was added till acidic reaction. The obtained precipitation was filtered out, washed with water, and purified by crystallization from methanol. It was obtained 3.64 g of **3e** (47 % yield), white crystalline solid, m.p. 268–270 °C; ^1^H NMR (DMSO-*d*_6_, 300 MHz,): *δ* = 10.83 (s, 1H, OH), 7.09–7.89 (m, 7H, CH_arom_), 4.05 (dd, 2H, *J* = 9.0, *J*′ = 7.3 Hz, H_2_-2), 4.18 (dd, 2H, *J* = 9.0, *J*′ = 7.3 Hz, H_2_-2), 3.28 (s, 2H, CH_2benzyl_); ^13^C NMR (DMSO-*d*_6_, 75 MHz,): *δ* = 41.3 (CBz), 41.3 (C-2), 42.7 (C-3), 91.2 (C-6), 117.2, 118.5, 120.5, 125.8, 128.4, 128.7, 129.0, 130.8, 130.8, 153.3 (C-7), 162.3 (C-8a), 167.5 (C-5),; EIMS *m/z* 388.1 [M+H]^+^. HREIMS (*m/z*): 387.0958 [M^+^] (calcd. for C_19_H_14_Cl_2_N_3_O_2_ 387.2590); Anal. calcd. for C_19_H_14_Cl_2_N_3_O_2_:C, 58.29; H, 3.64; Cl 18.31; N, 10.85. Found C, 58.40; H, 3.72; Cl, 18.28; N, 10.80.

#### 6-Benzyl-1-(2,6-dichlorphenyl)-7-hydroxy-2,3-dihydroimidazo[1,2-a]pyrimidine-5(1*H*)-one (**3f**)

0.02 (6.18 g) mol of hydrobromide of 1-(2,6-dichlorphenyl)-4,5-dihydro-1*H*-imidazol-2-amine (**1f**), 0.02 (5.0 g) mol of diethyl 2-benzylmalonate (**2a**), 15 mL of 16.7 % solution of sodium methoxide and 60 mL of methanol were heated in a round-bottom flask equipped with a condenser and mechanic mixer in boiling for 8 h. The reaction mixture was then cooled down, and the solvent was distilled off. The resulted solid was dissolved in 100 mL of water, and 10 % solution of hydrochloric acid was added till acidic reaction. The obtained precipitation was filtered out, washed with water, and purified by crystallization from methanol. It was obtained 3.40 g of **3f** (44 % yield), white crystalline solid, m.p. 274–275 °C; ^1^H NMR (DMSO-*d*_6_, 300 MHz,): *δ* = 11.03 (s, 1H, OH), 7.29–7.99 (m, 7H, CH_arom_), 4.01 (dd, 2H, *J* = 9.1, *J*′ = 7.6 Hz, H_2_-2), 4.21 (dd, 2H, *J* = 9.1, *J*′ = 7.6 Hz, H_2_-2), 3.38 (s, 2H, CH_2benzyl_); ^13^C NMR (DMSO-*d*_6_, 75 MHz,): *δ* = 24.1 (CBz), 40.2 (C-2), 42.6 (C-3), 94.2 (C-6), 117.9, 118.2, 119.6, 119.7, 122.4, 123.0, 123.9, 130.1, 130.3, 133.3, 133.3; 152.5 (C-7), 162.6 (C-8a), 166.8 (C-5),; EIMS *m/z* 388.1 [M+H]^+^. HREIMS (*m/z*): 387.1462 [M^+^] (calcd. for C_19_H_14_Cl_2_N_3_O_2_ 387.2590); Anal. calcd. for C_19_H_14_Cl_2_N_3_O_2_: C, 58.29; H, 3.64; Cl, 18.31; N, 10.85. Found C, 58.26; H, 3.42; Cl, 18.24; N, 10.76.

#### 6-Benzyl-1-(2-methylphenyl)-7-hydroxy-2,3-dihydroimidazo[1,2-a]pyrimidine-5(1*H*)-one (**3g**)

0.02 mol (5.08 g) of hydrobromide of 1-(2-methylphenyl)-4,5-dihydro-1*H*-imidazol-2-amine (**1** **g**), 0.02 mol (5.0 g) of diethyl 2-benzylmalonate (**2a**), 15 mL of 16.7 % solution of sodium methoxide and 60 mL of methanol were heated in a round-bottom flask equipped with a condenser and mechanic mixer in boiling for 8 h. The reaction mixture was then cooled down, and the solvent was distilled off. The resulted solid was dissolved in 100 mL of water, and 10 % solution of hydrochloric acid was added till acidic reaction. The obtained precipitation was filtered out, washed with water, and purified by crystallization from methanol. It was obtained 3.53 g of **3g** (53 % yield), white crystalline solid, m.p. 276–277 °C; ^1^H NMR (DMSO-*d*_6_, 300 MHz,): *δ* = 10.95 (s, 1H, OH), 7.19–7.75 (m, 9H, CH_arom_), 4.04 (dd, 2H, *J* = 9.0, *J*′ = 7.5 Hz, H_2_-2), 4.19 (dd, 2H, *J* = 9.0, *J*′ = 7.5 Hz, H_2_-2), 3.51 (s, 2H, CH_2benzyl_), 2.62 (s, 3H, CH_3_); ^13^C NMR (75 MHz, DMSO-*d*_6_): *δ* = 18.3 (CH_3_), 27.9 (CBz), 39.7 (C-2); 46.3 (C-3), 81.0 (C-6); 118.7, 119.4, 120.5, 121.3, 121.9, 123.2; 124.4, 125.2, 126.1, 126.9, 153.9 (C-7), 162.6 (C-8a), 171.2 (C-5); EIMS *m/z* 333.4 [M+H]^+^. HREIMS (*m/z*): 334.1452 [M^+^] (calcd. for C_20_H_19_N_3_O_2_ 333.3960); Anal. calcd. for C_20_H_19_N_3_O_2_: C, 72.05; H, 5.74; N, 12.60. Found C, 72.14; H, 5.60; N, 12.58.

#### 6-Benzyl-1-(4-methylphenyl)-7-hydroxy-2,3-dihydroimidazo[1,2-a]pyrimidine-5(1*H*)-one (**3h**)

0.02 mol (5.08 g) of hydrobromide of 1-(4-methylphenyl)-4,5-dihydro-1*H*-imidazol-2-amine (**1** **h**), 0.02 mol (5.0 g) of diethyl 2-benzylmalonate (**2a**), 15 mL of 16.7 % solution of sodium methoxide and 60 mL of methanol were heated in a round-bottom flask equipped with a condenser and mechanic mixer in boiling for 8 h. The reaction mixture was then cooled down, and the solvent was distilled off. The resulted solid was dissolved in 100 mL of water, and 10 % solution of hydrochloric acid was added till acidic reaction. The obtained precipitation was filtered out, washed with water, and purified by crystallization from methanol. It was obtained 3.00 g of **3** **h** (45 % yield), white crystalline solid, m.p. 300–302 °C; ^1^H NMR (DMSO-*d*_6_, 300 MHz,): *δ* = 10.98 (s, 1H, OH), 7.00–7.95 (m, 9H, CH_arom_), 4.00 (dd, 2H, *J* = 8.9, *J*′ = 7.4 Hz, H_2_-2), 4.16 (dd, 2H, *J* = 8.9, *J*′ = 7.4 Hz, H_2_-2), 3.63 (s, 2H, CH_2benzyl_), 2.32 (s, 3H, CH_3_); ^13^C NMR (DMSO-*d*_6_, 75 MHz,): *δ* = 18.0 (CH_3_), 28.2 (CBz), 41.5 (C-2), 48.3 (C-3), 91.9 (C-6), 123.2; 125.7, 127.6, 128.3, 128.3, 128.6, 128.7, 131.5, 137.0, 137.6; 153.9 (C-7), 162.7 (C-8a), 167.8 (C-5),; EIMS *m/z* 333.4 [M+H]^+^. HREIMS (*m/z*): 334.0972 [M^+^] (calcd. for C_20_H_19_N_3_O 333.3960); Anal. calcd. for C_20_H_19_N_3_O: C, 72.05; H, 5.74; N, 12.60. Found C, 71.44; H, 5.87; N, 12.53.

#### 6-Benzyl-1-(2,3-dimethylphenyl)-7-hydroxy-2,3-dihydroimidazo[1,2-a]pyrimidine-5(1*H*)-one (**3i**)

0.02 mol (5.36 g) of hydrobromide of 1-(2,3-dimethylphenyl)-4,5-dihydro-1*H*-imidazol-2-amine (**1i**), 0.02 mol (5.0 g) of diethyl 2-benzylmalonate (**2a**), 15 mL of 16.7 % solution of sodium methoxide and 60 mL of methanol were heated in a round-bottom flask equipped with a condenser and mechanic mixer in boiling for 8 h. The reaction mixture was then cooled down, and the solvent was distilled off. The resulted solid was dissolved in 100 mL of water, and 10 % solution of hydrochloric acid was added till acidic reaction. The obtained precipitation was filtered out, washed with water, and purified by crystallization from methanol. It was obtained 2.80 g of **3i** (44 % yield), white crystalline solid, m.p. 253–255 °C; ^1^H NMR (DMSO-*d*_6_, 300 MHz,): *δ* = 11.08 (s, 1H, OH), 7.20–7.80 (m, 8H, CH_arom_), 4.03 (dd, 2H, *J* = 9.1, *J*′ = 7.5 Hz, H_2_-2), 4.19 (dd, 2H, *J* = 9.1, *J*′ = 7.5 Hz, H_2_-2), 3.45 (s, 2H, CH_2benzyl_), 2.62 (s, 3H, CH_3_), 2.22 (s, 3H, CH_3_); ^13^C NMR (DMSO-*d*_6_, 75 MHz,): *δ* = 13.1 (CH_3_), 14.6 (CH_3_), 29.6 (CBz), 41.4 (C-2), 41.4 (C-3), 92.6 (C-6), 118.6, 120.3, 123.7, 124.9, 125.3, 126.6, 126.9, 128.3, 128.5, 129.7, 148.5 (C-7), 162.9 (C-8a), 168.9 (C-5),; EIMS *m/z* 347.1 [M+H]^+^. HREIMS (*m/z*): 348.1767[M^+^] (calcd. for C_21_H_21_N_3_O_2_ 347.4230); Anal. Found C, 72.43; H, 6.12; N, 12.00. calcd. C, 72.61; H, 6.09; N, 12.10.

#### 6-Benzyl-1-(2-methoxyphenyl)-7-hydroxy-2,3-dihydroimidazo[1,2-a]pyrimidine-5(1*H*)-one (**3j**)

0.02 mol (5.40 g) of hydrobromide of 1-(2-methoxyphenyl)-4,5-dihydro-1*H*-imidazol-2-amine (**1j**), 0.02 mol (5.0 g) of diethyl 2-benzylmalonate (**2a**), 15 mL of 16.7 % solution of sodium methoxide and 60 mL of methanol were heated in a round-bottom flask equipped with a condenser and mechanic mixer in boiling for 8 h. The reaction mixture was then cooled down, and the solvent was distilled off. The resulted solid was dissolved in 100 mL of water, and 10 % solution of hydrochloric acid was added till acidic reaction. The obtained precipitation was filtered out, washed with water, and purified by crystallization from methanol. It was obtained 4.47 g of **3j** (64 % yield), white crystalline solid, m.p. 258–260 °C; ^1^H NMR (DMSO-*d*_6_, 300 MHz,): *δ* = 10.78 (s, 1H, OH), 7.10–7.65 (m, 9H, CH_arom_), 4.06 (dd, 2H, *J* = 9.0, *J*′ = 7.6 Hz, H_2_-2), 4.20 (dd, 2H, *J* = 9.0, *J*′ = 7.6 Hz, H_2_-2), 3.25 (s, 2H, CH_2benzyl_), 2.12 (s, 3H, OCH_3_); ^13^C NMR (DMSO-*d*_6_, 75 MHz,): *δ* = 21.4 (OCH_3_), 28.9 (CBz), 40.2 (C-2), 45.3 (C-3), 90.4 (C-6), 118.7, 119.4, 120.1, 120.4, 121.3, 121.9, 123.2, 124.6, 125.6, 126.1;126.6, 154.7 (C-7), 158.2 (C-8a), 166.2 (C-5); EIMS *m/z* 349.1 [M+H]^+^. HREIMS (*m/z*): 350.1470[M^+^] (calcd. for C_20_H_19_N_3_O_3_ 349.3960); Anal. calcd. for C_20_H_19_N_3_O_3_: C, 68.75; H, 5.48; N, 12.03. Found C, 68.54; H, 5.29; N, 12.05.

#### 6-Benzyl-1-(4-metoxyphenyl)-7-hydroxy-2,3-dihydroimidazo[1,2-a]pyrimidine-5(1*H*)-one (**3k**)

0.02 mol (5.40 g) of hydrobromide of 1-(4-methoxyphenyl)-4,5-dihydro-1*H*-imidazol-2-amine (**1k**), 0.02 mol (5.0 g) of diethyl 2-benzylmalonate (**2a**), 15 mL of 16.7 % solution of sodium methoxide and 60 mL of methanol were heated in a round-bottom flask equipped with a condenser and mechanic mixer in boiling for 8 h. The reaction mixture was then cooled down, and the solvent was distilled off. The resulted solid was dissolved in 100 mL of water, and 10 % solution of hydrochloric acid was added till acidic reaction. The obtained precipitation was filtered out, washed with water, and purified by crystallization from methanol. It was obtained 4.47 g of **3k** (64 % yield), white crystalline solid, m.p. 298–300 °C; ^1^H NMR (DMSO-*d*_6_, 300 MHz,): *δ* = 10.65 (s, 1H, OH), 7.25–7.70 (m, 9H, CH_arom_), 4.03 (dd, 2H, *J* = 8.9, *J*′ = 7.4 Hz, H_2_-2), 4.19 (dd, 2H, *J* = 8.9, *J*′ = 7.4 Hz, H_2_-2), 3.56 (s, 2H, CH_2benzyl_), 2.82 (s, 3H, OCH_3_); ^13^C NMR (DMSO-*d*_6_, 75 MHz,): *δ* = 22.5 (OCH_3_), 29.1 (CBz), 40.5 (C-2), 46.3 (C-3), 90.8 (C-6), 120.3, 120.7, 122.0, 122.5, 123.1, 124.5, 125.6, 126.6, 126.8, 127.9, 155.1 (C-7), 156.1 (C-8a), 166.9 (C-5),; EIMS *m/z* 350.1 [M+H]^+^. HREIMS (*m/z*): 349.1767 [M^+^] (calcd. for C_20_H_19_N_3_O_3_ 349.3960); Anal. calcd. for C_20_H_19_N_3_O_3_: C, 68.75; H, 5.48; N, 12.03. Found C, 68.40; H, 5.66; N, 12.07.

#### 1,6-Dibenzyl-7-hydroxy-2,3-dihydroimidazo[1,2-a]pyrimidine-5(1*H*)-one, (**3l**)

0.02 mol (5.08 g) of hydrobromide of 1-benzyl-4,5-dihydro-1*H*-imidazol-2-amine (**1** **l**), 0.02 mol (5.0 g) of diethyl 2-benzylmalonate (**2a**), 15 mL of 16.7 % solution of sodium methoxide and 60 mL of methanol were heated in a round-bottom flask equipped with a condenser and mechanic mixer in boiling for 8 h. The reaction mixture was then cooled down, and the solvent was distilled off. The resulted solid was dissolved in 100 mL of water, and 10 % solution of hydrochloric acid was added till acidic reaction. The obtained precipitation was filtered out, washed with water, and purified by crystallization from methanol. It was obtained 3.13 g of **3l** (47 % yield), white crystalline solid, m.p. 234–236 °C; ^1^H NMR (DMSO-*d*_6_, 300 MHz,) *δ* = 10.80 (s, 1H, OH), 7.05–7.42 (m, 10H, CH_arom_), 3.51 (dd, 2H, *J* = 9.0, *J*′ = 7.6 Hz, H_2_-2), 3.96 (dd, 2H, *J* = 9.0, *J*′ = 7.6 Hz, H_2_-2), 3.49 (s, 2H, CH_2benzyl_), 4.53 (s, 2H, CH_2benzyl_), ^13^C NMR (DMSO-*d*_6_, 75 MHz,): *δ* = 26.0 (CBz), 28.6 (CBz), 41.1 (C-2), 44.8 (C-3), 91.4 (C-6), 111.4, 112.2, 112.5, 122.1, 125.8, 128.9, 128.3, 128.6, 129.2, 142.8 (C-7), 162.6 (C-8a), 167.6 (C-5),; EIMS *m/z* 334.1 [M+H]^+^. HREIMS (*m/z*): 333.1517 [M^+^] (calcd. for C_20_H_19_N_3_O_2_ 333.3960); Anal. calcd. for C_20_H_19_N_3_O_2_: C, 75.02; H, 5.74; N, 12.60. Found C, 75.27; H, 5.60; N, 12.56.

#### 6-(2-Chlorbenzyl)-1-phenyl-7-hydroxy-2,3-dihydroimidazo[1,2-a]pyrimidine-5(1*H*)-one (**3m**)

0.02 mol (4.84 g) of hydrobromide of 1-phenyl-4,5-dihydro-1*H*-imidazol-2-amine (**1a**), 0.02 mol (5.69 g) of diethyl 2-(2-chlorobenzyl)malonate (**2b**), 15 mL of 16.7 % solution of sodium methoxide and 60 mL of methanol were heated in a round-bottom flask equipped with a condenser and mechanic mixer in boiling for 8 h. The reaction mixture was then cooled down, and the solvent was distilled off. The resulted solid was dissolved in 100 mL of water, and 10 % solution of hydrochloric acid was added till acidic reaction. The obtained precipitation was filtered out, washed with water, and purified by crystallization from methanol. It was obtained 3.82 g of **3m** (54 % yield), white crystalline solid, m.p. 269–270 °C; ^1^H NMR (DMSO-*d*_6_, 300 MHz,): *δ* = 10.99 (s, 1H, OH), 7.06–7.86 (m, 9H, CH_arom_), 4.04 (dd, 2H, *J* = 9.0, *J*′ = 7.4 Hz, H_2_-2), 4.21 (dd, 2H, *J* = 9.0, *J*′ = 7.4 Hz, H_2_-2), 3.66 (s, 2H, CH_2benzyl_); ^13^C NMR (DMSO-*d*_6_, 75 MHz,): *δ* = 26.2 (CBz); 40.4 (C-2), 45.7 (C-3), 90.0 (C-6), 119.3, 123.7, 127.3, 127.71, 129.2, 129.3, 129.4, 133,5, 152.3 (C-7), 162.5 (C-8a), 167.6 (C-5),; EIMS *m/z* 354.8 [M+H]^+^. HREIMS (*m/z*) 353.1078 [M^+^] (calcd. for C_19_H_16_ClN_3_O_2_ 353.8180); Anal. calcd. for C_19_H_16_ClN_3_O_2_: C, 64.50; H, 4.56; Cl, 10.02; N, 11.88. Found C, 64.23; H, 4.70; Cl, 10.43; N, 11.70.

#### 6-(2-Chlorbenzyl)-1-(2-chlorphenyl)-7-hydroxy-2,3-dihydroimidazo[1,2-a]pyrimidine-5(1*H*)-one (**3n**)

0.02 mol (5.49 g) of hydrobromide of 1-(2-chlorphenyl)-4,5-dihydro-1*H*-imidazol-2-amine (**1b**), 0.02 mol (5.69 g) of diethyl 2-(2-chlorobenzyl)malonate (**2b**), 15 mL of 16.7 % solution of sodium methoxide and 60 mL of methanol were heated in a round-bottom flask equipped with a condenser and mechanic mixer in boiling for 8 h. The reaction mixture was then cooled down, and the solvent was distilled off. The resulted solid was dissolved in 100 mL of water, and 10 % solution of hydrochloric acid was added till acidic reaction. The obtained precipitation was filtered out, washed with water, and purified by crystallization from methanol. It was obtained 2.80 g of **3n** (44 % yield), white crystalline solid, m.p. 183–184 °C; ^1^H NMR (DMSO-*d*_6_, 300 MHz,): *δ* = 10.01 (s, 1H, OH), 7.15–7.96 (m, 8H, CH_arom_), 4.06 (dd, 2H, *J* = 9.0, *J*′ = 7.6 Hz, H_2_-2), 4.22 (dd, 2H, *J* = 9.0, *J*′ = 7.6 Hz, H_2_-2), 3.56 (s, 2H, CH_2benzyl_); ^13^C NMR (DMSO-*d*_6_, 75 MHz,): *δ* = 23.5 (CBz), 38.5 (C-2), 42.9 (C-3), 90.4 (C-6), 111.4, 116.9, 118.2, 127.3, 128.5, 128.8, 129.7, 131.6, 133.7, 136.6, 154.4 (C-7), 161.5 (C-8a), 169.5 (C-5),; EIMS *m/z* 389.1 [M+H]^+^. HREIMS (*m/z*) 388.0897 [M^+^] (calcd. for C_19_H_15_Cl_2_N_3_O_2_ 388.2670); Anal. calcd. for C_19_H_15_Cl_2_N_3_O_2_: C, 58.78; H, 3.90; Cl, 18.26; N, 10.82. Found C, 58.76; H, 3.83; Cl, 18.35; N, 10.80.

#### 6-(2-Chlorbenzyl)-1-(3-chlorphenyl)-7-hydroxy-2,3-dihydroimidazo[1,2-a]pyrimidine-5(1*H*)-one (**3o**)

0.02 mol (5.49 g) of hydrobromide of 1-93-chlorphenyl)-4,5-dihydro-1*H*-imidazol-2-amine (**1c**), 0.02 mol (5.69 g) of diethyl 2-(2-chlorobenzyl)malonate (**2b**), 15 mL of 16.7 % solution of sodium methoxide and 60 mL of methanol were heated in a round-bottom flask equipped with a condenser and mechanic mixer in boiling for 8 h. The reaction mixture was then cooled down, and the solvent was distilled off. The resulted solid was dissolved in 100 mL of water, and 10 % solution of hydrochloric acid was added till acidic reaction. The obtained precipitation was filtered out, washed with water, and purified by crystallization from methanol. It was obtained 5.98 g of **3o** (77 % yield), white crystalline solid, m.p. 272–274 °C; ^1^H NMR (300 MHz, DMSO-*d*_6_): *δ* = 11.12 (s, 1H, OH), 7.08–8.10 (m, 8H, CH_arom_), 4.05 (dd, 2H, *J* = 9.1, *J*′ = 7.6 Hz, H_2_-2), 4.16 (dd, 2H, *J* = 9.1, *J*′ = 7.6 Hz, H_2_-2), 3.68 (s, 2H, CH_2benzyl_); ^13^C NMR (75 MHz, DMSO-*d*_6_): *δ* = 26.2 (CBz), 40.4 (C-2), 45.6 (C-3), 90.6 (C-6), 117.2, 118.6, 123.2, 127.3, 127.7, 129.2, 130.1, 133.6, 133.9, 151.14 (C-7), 162.41 (C-8a), 167.53 (C-5),; EIMS *m/z* 389.1 [M+H]^+^. HREIMS (*m/z*) 388.0649 [M^+^] (calcd. for C_19_H_15_Cl_2_N_3_O_2_ 388.2670); Anal. calcd. for C_19_H_15_Cl_2_N_3_O_2_: C, 58.78; H, 3.90; Cl, 18.26; N, 10.82. Found C, 58.56; H, 3.92; Cl, 18.26; N, 10.86.

#### 6-(2-Chlorbenzyl)-1-(4-chlorphenyl)-7-hydroxy-2,3-dihydroimidazo[1,2-a]pyrimidine-5(1*H*)-one (**3p**)

0.02 mol (5.49 g) of hydrobromide of 1-(4-chlorphrnyl)-4,5-dihydro-1*H*-imidazol-2-amine (**1d**), 0.02 mol (5.69 g) of diethyl 2-(2-chlorobenzyl)malonate (**2b**), 15 mL of 16.7 % solution of sodium methoxide and 60 mL of methanol were heated in a round-bottom flask equipped with a condenser and mechanic mixer in boiling for 8 h. The reaction mixture was then cooled down, and the solvent was distilled off. The resulted solid was dissolved in 100 mL of water, and 10 % solution of hydrochloric acid was added till acidic reaction. The obtained precipitation was filtered out, washed with water, and purified by crystallization from methanol. It was obtained 6.99 g of **3p** (90 % yield), white crystalline solid, m.p. 288–290 °C; ^1^H NMR (DMSO-*d*_6_, 300 MHz,): *δ* = 10.51 (s, 1H, OH), 7.15–7.76 (m, 8H, CH_arom_), 4.02 (dd, 2H, *J* = 9.0, *J*′ = 7.6 Hz, H_2_-2), 4.19 (dd, 2H, *J* = 9.0, *J*′ = 7.6 Hz, H_2_-2), 3.56 (s, 2H, CH_2benzyl_); ^13^C NMR (DMSO-*d*_6_, 75 MHz,): *δ* = 23.23 (CBz), 40.2 (C-2), 45.9 (C-3), 90.4 (C-6), 120.4, 123.3, 125.7, 125.9, 126.7, 128.5, 129.2, 130.7, 131.5, 144.4 (C7), 161.5 (C-8a), 169.5 (C-5),; EIMS *m/z* 389.1 [M+H]^+^. HREIMS (*m/z*) 388.1766 [M^+^] (calcd. for C_19_H_15_Cl_2_N_3_O_2_ 388.2670); Anal. calcd. for C_19_H_15_Cl_2_N_3_O_2_: C, 58.78; H, 3.90; Cl, 18.26; N, 10.82. Found C, 58.45; H, 3.94; Cl, 18.27; N, 10.80.

#### 6-(2-Chlorbenzyl)-1-(3,4-dichlorphenyl)-7-hydroxy-2,3-dihydroimidazo[1,2-a]pyrimidine-5(1*H*)-one (**3q**)

0.02 mol (6.18 g) of hydrobromide of 1-(3,4-dichlorphenyl)-4,5-dihydro-1*H*-imidazol-2-amine (**1e**), 0.02 mol (5.69 g) of diethyl 2-(2-chlorobenzyl)malonate (**2b**), 15 mL of 16.7 % solution of sodium methoxide and 60 mL of methanol were heated in a round-bottom flask equipped with a condenser and mechanic mixer in boiling for 8 h. The reaction mixture was then cooled down, and the solvent was distilled off. The resulted solid was dissolved in 100 mL of water, and 10 % solution of hydrochloric acid was added till acidic reaction. The obtained precipitation was filtered out, washed with water, and purified by crystallization from methanol. It was obtained 2.78 g of **3q** (32 % yield), white crystalline solid, m.p. 222–224 °C; ^1^H NMR (DMSO-*d*_6_, 300 MHz,): *δ* = 11.01 (s, 1H, OH) 7.05–7.65 (m, 7H, CH_arom_), 4.05 (dd, 2H, *J* = 9.1, *J*′ = 7.6 Hz, H_2_-2), 4.20 (dd, 2H, *J* = 9.1, *J*′ = 7.6 Hz, H_2_-2), 3.46 (s, 2H, CH_2benzyl_); ^13^C NMR (DMSO-*d*_6_, 75 MHz,): *δ* = 25.9 (CBz), 39.9 (C-2), 45.4 (C-3), 92.4 (C-6), 120.3, 123.5, 125.2, 126.9, 127.3, 128.2, 131.1, 131.6, 132.2, 132.6, 154.1 (C-7), 161.1 (C-8a), 164.5 (C-5),; EIMS *m/z* 423.7 [M+H]^+^. HREIMS (*m/z*) 422.2516 [M^+^] (calcd. for C_19_H_14_Cl_3_N_3_O_2_ 422.7160); Anal. calcd. for C_19_H_14_Cl_3_N_3_O_2_: C, 53.99; H, 3.34; Cl, 25.16; N, 9.94. Found C, 54.15; H, 3.94; Cl, 24.97; N, 9.96.

#### 6-(2-Chlorbenzyl)-1-(2,6-dichlorphenyl)-7-hydroxy-2,3-dihydroimidazo[1,2-a]pyrimidine-5(1*H*)-one (**3r**)

0.02 mol (6.18 g) of hydrobromide of 1-(2,6-dichlorphenyl)-4,5-dihydro-1*H*-imidazol-2-amine (**1f**), 0.02 mol (5.69 g) of diethyl 2-(2-chlorobenzyl)malonate (**2b**), 15 mL of 16.7 % solution of sodium methoxide and 60 mL of methanol were heated in a round-bottom flask equipped with a condenser and mechanic mixer in boiling for 8 h. The reaction mixture was then cooled down, and the solvent was distilled off. The resulted solid was dissolved in 100 mL of water, and 10 % solution of hydrochloric acid was added till acidic reaction. The obtained precipitation was filtered out, washed with water, and purified by crystallization from methanol. It was obtained 3.12 g of **3r** (37 % yield), white crystalline solid, m.p. 269–270 °C; ^1^H NMR (DMSO-*d*_6_, 300 MHz,): *δ* = 10.86 (s, 1H, OH); 7.25–7.70 (m, 7H, CH_arom_); 4.03 (dd, 2H, *J* = 9.0, *J*′ = 7.5 Hz, H_2_-2), 4.19 (dd, 2H, *J* = 9.0, *J*′ = 7.5 Hz, H_2_-2), 3.16 (s, 2H, CH_2benzyl_); ^13^C NMR (DMSO-*d*_6_, 75 MHz,): *δ* = 26.3 (CBz), 40.1 (C-2), 46.0 (C-3), 90.1 (C-6), 118.7, 121.8, 122.2, 123.3, 124.4, 125.6, 126.5, 126.8, 127.9, 128.1, 130.3, 131.2, 154.2 (C-7), 160.1 (C-8a), 165.5 (C-5),; EIMS *m/z* 423.7 [M+H]^+^. HREIMS (*m/z*) 422.1228 [M^+^] (calcd. C_19_H_14_Cl_3_N_3_O_2_ 422.7160); Anal. calcd. for C_19_H_14_Cl_3_N_3_O_2_: C, 53.99; H, 3.34; Cl, 25.16; N, 9.94. Found C, 53.84; H, 3.20; Cl, 24.73; N, 9.90.

#### 6-(2-Chlorbenzyl)-1-(2-methylphenyl)-7-hydroxy-2,3-dihydroimidazo[1,2-a]pyrimidine-5(1*H*)-one (**3s**)

0.02 mol (5.08 g) of hydrobromide of 1-(2-methylphenyl)-4,5-dihydro-1*H*-imidazol-2-amine (**1g**), 0.02 mol (5.69 g) of diethyl 2-(2-chlorobenzyl)malonate (**2b**), 15 mL of 16.7 % solution of sodium methoxide and 60 mL of methanol were heated in a round-bottom flask equipped with a condenser and mechanic mixer in boiling for 8 h. The reaction mixture was then cooled down, and the solvent was distilled off. The resulted solid was dissolved in 100 mL of water, and 10 % solution of hydrochloric acid was added till acidic reaction. The obtained precipitation was filtered out, washed with water, and purified by crystallization from methanol. It was obtained 5.22 g of **3** **s** (71 % yield), white crystalline solid, m.p. 280–281 °C; ^1^H NMR (DMSO-*d*_6_, 300 MHz,): *δ* = 10.93 (s, 1H, OH), 7.06–7.73 (m, 8H, CH_arom_), 4.05 (dd, 2H, *J* = 9.0, *J*′ = 7.6 Hz, H_2_-2), 4.17 (dd, 2H, *J* = 9.0, *J*′ = 7.6 Hz, H_2_-2), 3.66 (s, 2H, CH_2benzyl_), 2.32 (s, 3H, CH_3_); ^13^C NMR (DMSO-*d*_6_, 75 MHz,) *δ* = 20.7 (CH_3_), 26.2 (CBz), 41.1 (C-2), 45.2 (C-3), 90.1 (C-6), 119.4, 120.1, 120.5, 121.2, 122.9, 123.2, 125.6, 125.8;, 128.6, 128.8, 129.4, 130.3, 152.6 (C-7), 162.9 (C-8a), 166.6 (C-5);, EIMS *m/z* 368.2 [M+H]^+^. HREIMS (*m/z*) 367.2516 [M^+^] (calcd. for C_20_H_18_ClN_3_O_2_ 367.8450),; Anal. calcd. for C_20_H_18_ClN_3_O_2_: C, 65.30; H, 4.93; Cl, 9.64; N, 11.42. Found C, 64.66; H, 4.85; Cl, 9.92; N, 11.40.

#### 6-(2-Chlorbenzyl)-1-(4-methylphenyl)-7-hydroxy-2,3-dihydroimidazo[1,2-a]pyrimidine-5(1*H*)-one (**3t**)

0.02 mol (5.08 g) of hydrobromide of 1-(4-methylphenyl)-4,5-dihydro-1*H*-imidazol-2-amine (**1h**), 0.02 mol (5.69 g) of diethyl 2-(2-chlorobenzyl)malonate (**2b**), 15 mL of 16.7 % solution of sodium methoxide and 60 mL of methanol were heated in a round-bottom flask equipped with a condenser and mechanic mixer in boiling for 8 h. The reaction mixture was then cooled down, and the solvent was distilled off. The resulted solid was dissolved in 100 mL of water, and 10 % solution of hydrochloric acid was added till acidic reaction. The obtained precipitation was filtered out, washed with water, and purified by crystallization from methanol. It was obtained 4.93 g of **3t** (67 % yield), white crystalline solid, m.p. 300–302 °C; ^1^H NMR (300 MHz, DMSO-*d*_6_): *δ* = 10.93 (s, 1H, OH), 7.05–7.65 (m, 8H, CH_arom_), 4.05 (dd, 2H, *J* = 9.0, *J*′ = 7.5 Hz, H_2_-2), 4.15 (dd, 2H, *J* = 8.9, *J*′ = 7.5 Hz, H_2_-2), 3.40 (s, 2H, CH_2benzyl_), 2.32 (s, 3H, CH_3_); ^13^C NMR (DMSO-*d*_6_, 75 MHz,): *δ* = 20.9 (CH_3_), 26.2 (CBz), 40.4 (C-2), 45.9 (C-3), 89.8 (C-6), 119.7, 127.3, 127.7, 129.2, 129.4, 129.7, 133.1, 133.5, 137.3, 138.7, 152.4 (C-7), 162.6 (C-8a), 167.6 (C-5),; EIMS *m/z* 368.8 [M+H]^+^. HREIMS (*m/z*) 367.1219 [M^+^] (calcd. for C_20_H_18_ClN_3_O_2_ 367.8450); Anal. calcd. for C_20_H_18_ClN_3_O_2_: C, 65.30; H, 4.93; Cl, 9.64; N, 11.42. Found C, 65.32; H, 4.85; Cl, 9.10; N, 11.46.

#### 6-(2-Chlorbenzyl)-1-(2,3-dimethylphenyl)-7-hydroxy-2,3-dihydroimidazo[1,2-a]pyrimidine-5(1*H*)-one (**3u**)

0.02 mol (5.36 g) of hydrobromide of 1-(2,3-dimethylphenyl)-4,5-dihydro-1*H*-imidazol-2-amine (**1i**), 0.02 mol (5.69 g) of diethyl 2-(2-chlorobenzyl)malonate (**2b**), 15 mL of 16.7 % solution of sodium methoxide and 60 mL of methanol were heated in a round-bottom flask equipped with a condenser and mechanic mixer in boiling for 8 h. The reaction mixture was then cooled down, and the solvent was distilled off. The resulted solid was dissolved in 100 mL of water, and 10 % solution of hydrochloric acid was added till acidic reaction. The obtained precipitation was filtered out, washed with water, and purified by crystallization from methanol. It was obtained 2.29 g of **3u** (30 % yield), white crystalline solid, m.p. 223–225 °C; ^1^H NMR (DMSO-*d*_6_, 300 MHz,): *δ* = 10.68 (s, 1H, OH), 7.06–7.73 (m, 7H, CH_arom_), 4.01 (dd, 2H, *J* = 9.1, *J*′ = 7.4 Hz, H_2_-2), 4.19 (dd, 2H, *J* = 9.1, *J*′ = 7.4 Hz, H_2_-2), 3.66 (s, 2H, CH_2benzyl_), 2.32 (s, 3H, CH_3_), 2.02 (s, 3H, CH_3_) ^13^C NMR (DMSO-*d*_6_, 75 MHz,): *δ* = 19.5 (CH_3_), 20.8 (CH_3_), 26.2 (CBz), 40.4 (C-2), 45.9 (C-3), 89.8 (C-6), 120.9, 121.3, 121.9, 123.4, 124.6, 125.2, 126.1, 128.3, 129.1, 131.2, 152.4 (C-7), 162.6 (C-8a), 167.7 (C-5),; EIMS *m/z* 382.2 [M+H]^+^. HREIMS (*m/z*) 381.2194 [M^+^] (calcd. for C_21_H_20_ClN_3_O_2_ 381.8720); Anal. calcd. for C_21_H_20_ClN_3_O_2_: C, 66.05; H, 5.28; Cl, 9.29;N, 11.00. Found C, 66.10; H, 5.20; Cl, 9.71; N, 10.83.

#### 6-(2-Chlorbenzyl)-1-(2-methoxyphenyl)-7-hydroxy-2,3-dihydroimidazo[1,2-a]pyrimidine-5(1*H*)-one (**3v**)

0.02 mol (5.40 g) of hydrobromide of 1-(2-methyoxyphenyl)-4,5-dihydro-1*H*-imidazol-2-amine (**1j**), 0.02 mol (5.69 g) of diethyl 2-(2-chlorobenzyl)malonate (**2b**), 15 mL of 16.7 % solution of sodium methoxide and 60 mL of methanol were heated in a round-bottom flask equipped with a condenser and mechanic mixer in boiling for 8 h. The reaction mixture was then cooled down, and the solvent was distilled off. The resulted solid was dissolved in 100 mL of water, and 10 % solution of hydrochloric acid was added till acidic reaction. The obtained precipitation was filtered out, washed with water, and purified by crystallization from methanol. It was obtained 4.84 g of **3v** (63 % yield), white crystalline solid, m.p. 257–258 °C; ^1^H NMR (DMSO-*d*_6_, 300 MHz,): *δ* = 10.63 (s, 1H, OH), 7.01–7.64 (m, 8H, CH_arom_), 4.00 (dd, 2H, *J* = 8.9, *J*′ = 7.5 Hz, H_2_-2), 4.15 (dd, 2H, *J* = 8.9, *J*′ = 7.5 Hz, H_2_-2), 3.65 (s, 2H, CH_2benzyl_), 2.52 (s, 3H, OCH_3_); ^13^C NMR (DMSO-*d*_6_, 75 MHz,): *δ* = 18.3 (OCH_3_), 28.5 (CBz), 42.5 (C-2), 48.3 (C-3), 91.6 (C-6), 119.33, 120.78, 121.55, 123.74, 127.48, 128.27, 128.34, 128.50, 128.74, 131.28; 153.2 (C-7), 162.7 (C-8a), 168.7 (C-5),; EIMS *m/z* 384.8 [M+H]^+^. HREIMS (*m/z*) 383.1542 [M^+^] (calcd. for C_20_H_18_ClN_3_O_3_ 383.8450); Anal. calcd. for C_20_H_18_ClN_3_O_3_: C, 62.58; H, 4.73; Cl, 9.24; N, 10.95. Found C, 62.40; H, 4.70; Cl, 9.33; N, 10.92.

#### 6-(2-Chlorbenzyl)-1-(4-methoxyphenyl)-7-hydroxy-2,3-dihydroimidazo[1,2-a]pyrimidine-5(1*H*)-one (**3w**)

0.02 mol (5.40 g) of hydrobromide of 1-(4-methoxyphenyl)-4,5-dihydro-1*H*-imidazol-2-amine (**1k**), 0.02 mol (5.69 g) of diethyl 2-(2-chlorobenzyl)malonate (**2b**), 15 mL of 16.7 % solution of sodium methoxide and 60 mL of methanol were heated in a round-bottom flask equipped with a condenser and mechanic mixer in boiling for 8 h. The reaction mixture was then cooled down, and the solvent was distilled off. The resulted solid was dissolved in 100 mL of water, and 10 % solution of hydrochloric acid was added till acidic reaction. The obtained precipitation was filtered out, washed with water, and purified by crystallization from methanol. It was obtained 3.45 g of **3w** (45 % yield), white crystalline solid, m.p. 278–279 °C; ^1^H NMR (DMSO-*d*_6_, 300 MHz,): *δ* = 11.09 (s, 1H, OH), 7.05–7.84 (m, 8H, CH_arom_), 4.02 (dd, 2H, *J* = 9.1 Hz, *J*′ = 7.6, H_2_-2), 4.18 (dd, 2H, *J* = 9.1 Hz, *J*′ = 7.6, H_2_-2), 3.85 (s, 2H, CH_2benzyl_), 3.05 (s, 3H, OCH_3_); ^13^C NMR (75 MHz, DMSO-*d*_6_): *δ* = 21.6 (OCH_3_), 24.5 (CBz), 41.2 (C-2), 44.3 (C-3), 90.6 (C-6), 119.5, 121.8, 121.1, 122.3, 123.9, 124.3, 129.3, 129.5, 131.7, 132.3; 153.9 (C-7), 162.5 (C-8a), 170.9 (C-5),; EIMS *m/z* 384.8 [M+H]^+^. HREIMS (*m/z*) 383.2533 [M^+^] (calcd. for C_20_H_18_ClN_3_O_3_ 383.8450); Anal. calcd. for C_20_H_18_ClN_3_O_3_: C, 62.58; H, 4.73; Cl, 9.24; N, 10.95. Found C, 62.43; H, 4.62; Cl, 9.34; N, 10.90.

#### 6-(2-Chlorbenzyl)-1-benzyl-7-hydroxy-2,3-dihydroimidazo[1,2-a]pyrimidine-5(1*H*)-one (**3x**)

0.02 mol (5.08 g) of hydrobromide of 1-(benzyl)-4,5-dihydro-1*H*-imidazol-2-amine (**1** **l**), 0.02 mol (5.69 g) of diethyl 2-(2-chlorobenzyl)malonate (**2b**), 15 mL of 16.7 % solution of sodium methoxide and 60 mL of methanol were heated in a round-bottom flask equipped with a condenser and mechanic mixer in boiling for 8 h. The reaction mixture was then cooled down, and the solvent was distilled off. The resulted solid was dissolved in 100 mL of water, and 10 % solution of hydrochloric acid was added till acidic reaction. The obtained precipitation was filtered out, washed with water, and purified by crystallization from methanol. It was obtained 5.38 g of **3x** (73 % yield), white crystalline solid, m.p. 259–260 °C; ^1^H NMR (DMSO-*d*_6_, 300 MHz,): *δ* = 10.97 (s, 1H, OH), 7.06–7.44 (m, 9H, CH_arom_), 3.58 (s, 2H, CH_2benzyl_), 3.94 (dd,2H, *J* = 8.9, *J*′ = 7.3 Hz, H_2_-2), 4.00 (dd,2H, *J* = 9.0, *J*′ = 7.3 Hz, H_2_-2), 3.62 (s, 2H, CH_2benzyl_); ^13^C NMR (DMSO-*d*_6_, 75 MHz,): *δ* = 26.2 (CBz), 41.1 (CBz), 44.5 (C-2), 47.8 (C-3), 88.3 (C-6), 127.3, 127.6, 128.1, 128.2, 129.1, 129.4, 129.2, 129.4, 133.5, 136.7, 155.2 (C-7), 162.7 (C-8a), 168.4 (C-5), EIMS *m/z* 368.8 [M+H]^+^. HREIMS (*m/z*) 367.1227 [M^+^] (calcd. for C_20_H_19_ClN_3_O_2_ 368.8530); Anal. calcd. for C_20_H_19_ClN_3_O_2_: C, 65.30; H, 4.93; Cl, 9.64; N, 11.42. Found C, 65.41; H, 5.15; Cl, 10.02; N, 11.50.

### Molecular modeling

The investigated compounds were modeled using the LigPrep protocol from the Schrödinger Suite (LigPrep version 2.4, 2010). In order to sample different protonation states of ligands in physiological pH, Epik module was used (Epik version 2.1, 2010). Parameters to estimate drug-likeness were calculated using VegaZZ (Pedretti *et al.*, 2004) (molar mass, number of atoms), Discovery Studio 3.1. (Discovery Studio 3.1, Accelrys) (number of rings, lipophilicity, number of rotatable bonds), ACDLabs (molar refractivity, number of hydrogen bond donors and acceptors), and MOE Molecular Environment (MOE Molecular Operating Environment 2009/2010) (a number of rigid bonds). ADMET parameters were calculated with Discovery Studio 3.1 (blood–brain permeation, solubility) or PREADMET service (Lee *et al.*, 2004) (human intestinal absorption). For structure–activity relationship studies, HOMO and LUMO energies were calculated with Discovery Studio 3.1. HOMO and LUMO orbitals as well as a map of the electrostatic potential (ESP) onto a surface of the electron density were visualized with ArgusLab (http://www.arguslab.com). Polar surface area, molar volume, and polarizability were calculated with ACDLabs software.

### Pharmacology

#### Behavioral tests

The experiments were performed on male Albino Swiss mice (20–25 g). The animals were kept 8–10 to a cage, at room temperature of 20 ± 1 °C, on a 12:12 h dark–light cycle. Standard food (laboratory pellets, Bacutil, Motycz, Poland) and water were available ad libitum. The experiments were performed between 8 a.m. and 3 p.m. and were performed in accordance with the opinion of Local Ethics Committee for Animal Experimentation. The investigated substances, marked as **3a**, **3d**, **3g**, **3l**, **3n**, **3p,** and **3s,** were administered intraperitoneally (i.p.) in volume of 10 cm^3^/kg as suspensions in *aqueous* solution of 0.5 % methylcellulose (tylose) in the doses equivalent to 0.1, 0.05, 0.025, 0.0125, and 0.00625 ED_50_. The compounds were injected 60 min before the tests. The controls received the equivalent volume of the solvent. All tests performed as suggested by Vogel and Vogel (Vogel and Vogel, 1997) are generally accepted as basic in investigation of the central activity by behavioral methods. The acute toxicity of the compound was assessed in mice acc. to Litchfield and Wilcoxon method (Litchfield and Wilcoxon, 1949) as the ED_50_ calculated on the loss of the righting reflex within 48 h.

In addition, the activity of the compounds was assessed in the following tests: (1) locomotor activity measured in photoresistor actometers for a single mouse for 30 min as spontaneous activity and amphetamine-induced hyperactivity **(**mice received subcutaneusly (s.c.) 5 mg/kg of amphetamine 30 min before the test); (2) nociceptive reactions studied in the acetic acid (0.6 %) induced writhing test (the number of writhing episodes was measured for 10 min starting 5 min after i.p. administration of acid solution); (3) motor coordination evaluated in the rota-rod test; (4) body temperature in normothermic mice measured in the rectum of animals with a thermistor thermometer; (5) pentylenetetrazole (110 mg/kg, s.c.)-induced convulsions were evaluated as the number of mice with clonic seizures, tonic convulsions, and dead animals; (6) head-twitch responses (HTR) after 5-hydroxytryptophan (L-5-HTP) recorded according to Corne et al. (1963) (mice received 5-HTP (230 mg/kg, i.p.) and the number of head-twitches was recorded in 6 two-minutes intervals (4–6, 14–16, 24–26, 34–36, 44–46, 54–56 min) during 1 h); (7) influence of naloxone (5 mg/kg, s.c.) on the antinociceptive effect of the compounds assessed in the writhing test.

#### Statystical analysis

The obtained data were calculated by χ^2^ test with Yates correction (PTZ-induced seizures) and one-way analysis of variance (ANOVA) (other tests). Post-hoc comparisons were carried out by means of Dunnett test. All results are presented in the figures as mean ± SEM. A probability (p) value of 0.05 or less was considered as statistically significant.

## Results and Discussion

### Chemistry

**Fig. 4:**
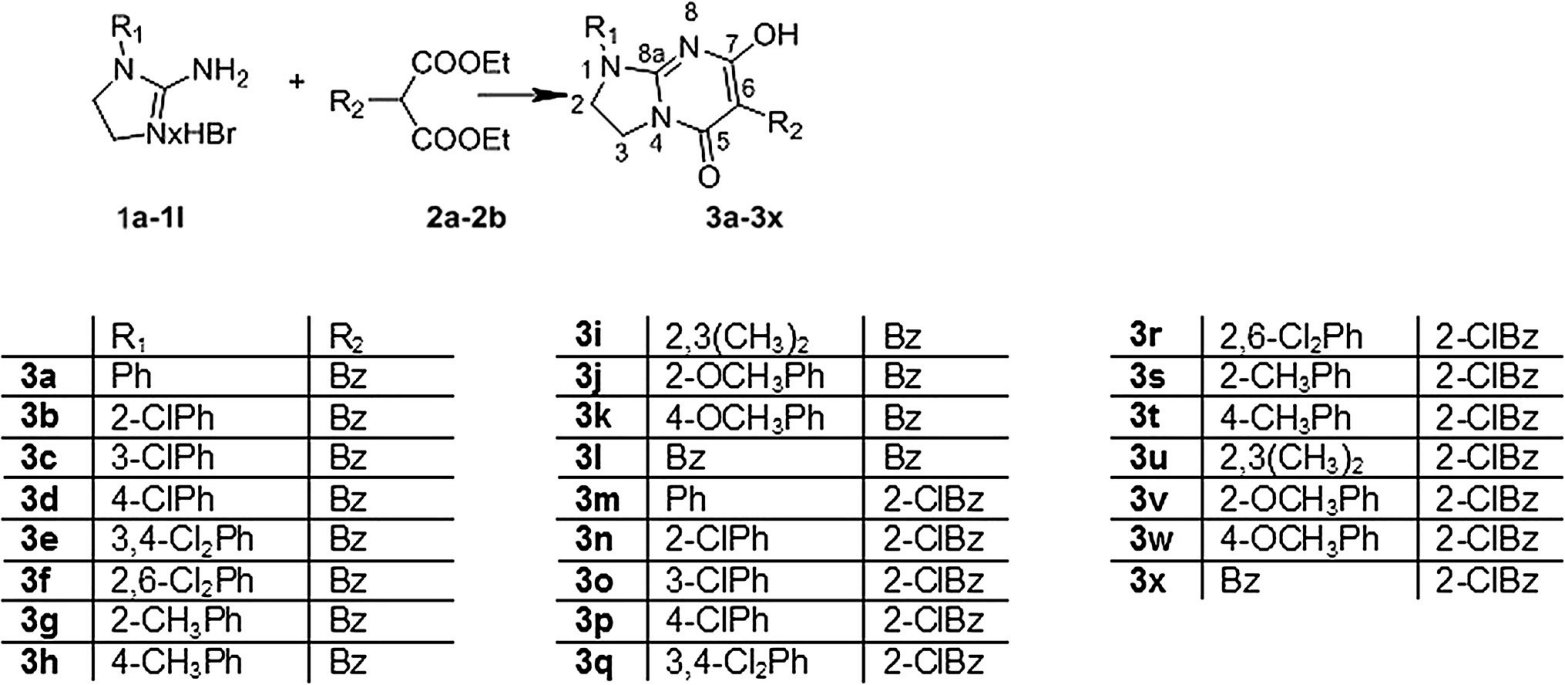
The scheme of synthesis of the investigated compounds

The compounds **3a**–**3x** were obtained in one-step cyclocondensation of 1-aryl-4,5-dihydro-1*H*-imidazol-2-amines (**1a**–**1l**) diethyl 2-benzylmalonate (**2a**) or diethyl 2-(2-chlorobenzyl)malonate (**2b**) under basic conditions (sodium methoxide), Fig. 4 cyclocondensation reaction. The cyclocondensation reaction of this type was earlier reported as a method of preparation of imidazo[1,2-a]pyrimidines (Matosiuk *et al.*, 1996) as well as other derivatives of 1-aryl-4,5-dihydro-1*H*-imidazol-2-amines (Matosiuk *et al*., 2002a, b; Sztanke *et al.*, 2005) and 1-aryl-4,5-dihydro-1*H*-imidazol-2-hydrazines (Sztanke, 2002, 2004). Reaction of imidazole-2-amines with electrophilic compounds represents one of the synthetic methods to build this heterocyclic system. The main alternative involves the imidazole ring closure by condensation of pyrimidin-2-amines with an appropriate compound.

### Estimation of drug-likeness

The descriptors used for estimation of drug-likeness are collected in Table 1. Drug-likness was assessed using Lipinski’s rule as well as the placement of the investigated compounds in the chemical space determined by the databases of the pharmacologically active compounds (CMC, Comprehensive Medicinal Chemistry Database, containing about 7,000 compounds and MDDR, MACCS-II Drug Data Report, containing about 100,000 compounds) according to the methodology of PREADMET service. Regarding Lipinski’s rule, all the compounds possess the molar mass below 500, the number of hydrogen bond donors below 5, the number of hydrogen bond acceptors below 10, and the lipohilicity below 5.

**Table 1:** Parameters for drug-likeness estimation

Comp.	Molar mass	Lipophilicity AlogP98	HBD	HBA	Number of atoms	Molar refractivity	Rings	Rigid bonds	Rotatable bonds
**3a**	319.36	2.766	1	5	41	92.58	4	41	3
**3b**	353.80	3.431	1	5	41	97.18	4	41	3
**3c**	353.80	3.431	1	5	41	97.18	4	41	3
**3d**	353.80	3.431	1	5	41	97.18	4	41	3
**3e**	388.24	4.095	1	5	41	101.78	4	41	3
**3f**	388.24	4.095	1	5	41	101.78	4	41	3
**3g**	333.38	3.252	1	5	44	97.00	4	44	3
**3h**	333.38	3.252	1	5	44	97.00	4	44	3
**3i**	347.41	3.739	1	5	47	101.43	4	47	3
**3j**	349.38	2.750	1	6	45	98.39	4	45	4
**3k**	349.38	2.750	1	6	45	98.39	4	44	4
**3l**	333.38	2.773	1	5	44	97.19	4	43	4
**3m**	353.80	3.431	1	5	41	97.18	4	40	3
**3n**	388.24	4.095	1	5	41	101.78	4	41	3
**3o**	388.24	4.095	1	5	41	101.78	4	41	3
**3p**	388.24	4.095	1	5	41	101.78	4	41	3
**3q**	422.69	4.759	1	5	41	106.38	4	41	3
**3r**	422.69	4.759	1	5	41	106.38	4	41	3
**3s**	367.83	3.917	1	5	44	101.60	4	44	3
**3t**	367.83	3.917	1	5	44	101.60	4	44	3
**3u**	381.86	4.403	1	5	47	106.03	4	47	3
**3v**	383.83	3.414	1	6	45	102.99	4	44	4
**3w**	383.83	3.414	1	6	45	102.99	4	44	4
**3x**	367.83	3.438	1	5	44	101.79	4	43	4

*HBD* a number of hydrogen bond donors, *HBA* a number of hydrogen bond acceptors

Concerning subsequent criteria of drug-likeness, most compounds collected in the CMC database has lipophilicity from -0.4 to 5.6, molar refractivity in the range of 40–130, molar mass from 160 to 480, and the number of atoms from 20 to 70. All the investigated compounds fulfill this criterion. In respect to the compounds in MDDR database, the drug-like substances have the number of rings equal or greater than 3, the number of rigid bonds equal or greater than 18, and the number of rotatable bonds equal or greater than 6. Thus, the investigated substances fulfill the first two conditions, but it may turn out favorable to increase the number of rotatable bonds which we will consider in the design of next series of compounds.

In conlusion, the investigated compounds may be termed drug-like, and it is justified to test them in the in vivo experiments.

### Prediction of ADMET properties

**Fig. 5:**
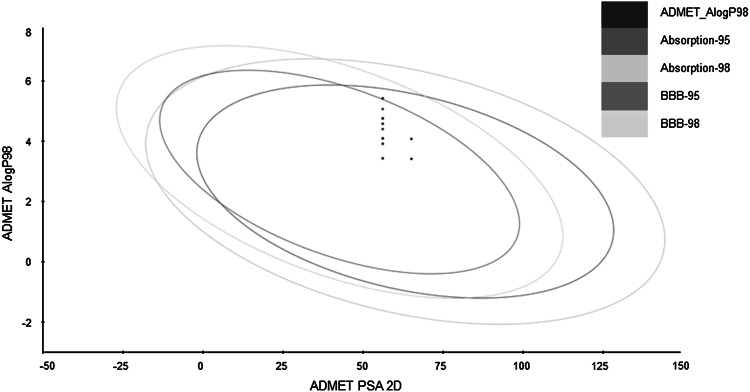
The plot of ADMET properties of the investigated compounds

In order to facilitate the selection of compounds for animal studies, some ADMET parameters were calculated (Table 2). In addition, all the tested compounds have human intestinal absorption of about 97 %. The plot presented in Fig. 5 confirms that most of the tested compounds possess favorable ADMET properties, although some of them have borderline values.

**Table 2:** ADMET parameters of the studied compounds

Compound	Log BBB	Log S
**3a**	0.018	−4.341
**3b**	0.223	−5.067
**3c**	0.223	−5.059
**3d**	0.223	−5.050
**3e**	0.428	−5.767
**3f**	0.428	−5.792
**3g**	0.168	−4.826
**3h**	0.168	−4.809
**3i**	0.318	−5.301
**3j**	−0.129	−4.382
**3k**	−0.129	−4.348
**3l**	0.02	−4.235
**3m**	0.223	−5.065
**3n**	0.428	−5.786
**3o**	0.428	−5.777
**3p**	0.428	−5.768
**3q**	0.634	−6.478
**3r**	0.634	−6.505
**3s**	0.373	−5.544
**3t**	0.373	−5.527
**3u**	0.524	−6.014
**3v**	0.077	−5.094
**3w**	0.077	−5.059
**3x**	0.225	−4.951

*BBB* blood–brain barrier, *S* solubility

On the basis of calculation of ADMET parameters, we decided to exclude compounds **3j** and **3k** from the set to animal studies. However, compound **3l** was included in this set, firstly, due to the structure originality and secondly, as a validation of ADMET parameter calculation.

### Pharmacology

Seven compounds were tested for their pharmacological activity. The compounds were selected for the pharmacological evaluation on the basis of the results for the previously reported series. They exhibited very low toxicity: over 2,000 mg/kg i.p.; therefore, ED_50_ = 2,000 mg/kg was accepted, and the regressive doses of 200, 100, 50, 25, and 12.5 mg/kg i.p of the tested compounds were used for further studies. The tested compounds are composed of two groups: **3a**, **3d**, **3g,** and **3l** possess the benzyl groups at C6 carbon atom, whereas **3n**, **3p**, and **3s** have 2-chlorobenzyl moiety at this atom.

From the group of the compounds tested, only **3l** was almost totally devoid of activity in the CNS. It showed only a weak, but significant (*p* < 0.05) inhibitory effect on locomotor activity of animals, in other tests performed remained inactive.

**Fig. 6:**
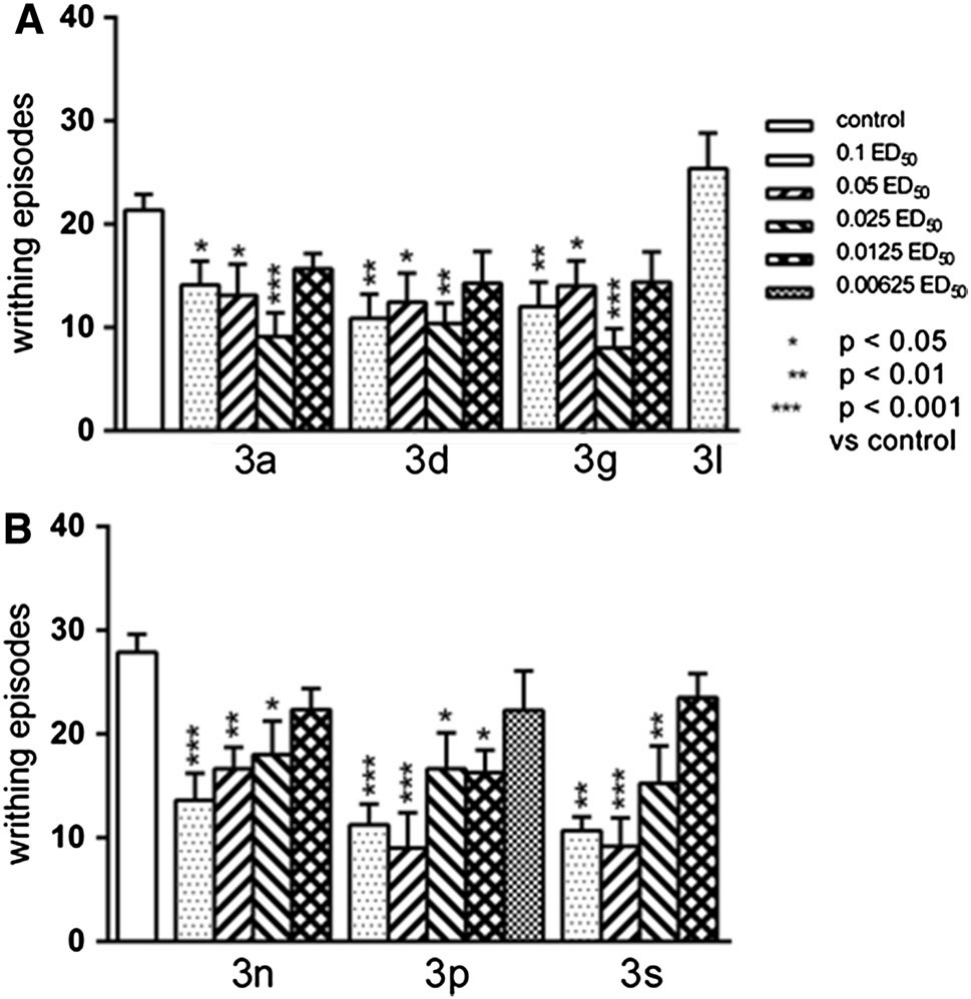
The antinociceptive effects of the tested compounds, assessed in the “writhing” test in mice. The results are expressed as mean ± SEM of a group of 8–18 mice. A—one-way ANOVA showed significant changes in the numer of writhing episodes of mice after the administration of the compound **3a** (*F*_4.43_ = 5.627, *p* = 0.001), **3d** (*F*_4.46_ = 5.537, *p* = 0.001), **3g** (*F*_4.47_ = 6.281, *p* < 0.001). Post-hoc Tukey’s test confirmed a significant reduction in the writhing episodes of mice after the administration of the compound **3a** in the dose of 0.1, 0.05 ED_50_ (*p* < 0.05), and 0.025 ED_50_ (*p* < 0.001), **3d**—0.1, 0.05, 0.025 ED_50_ (appropriately *p* < 0.01, *p* < 0.05, *p* < 0.01), **3g**—0.1, 0.05, 0.025 ED50 (*p* < 0.01, *p* < 0.05, *p* < 0.001). B—One-way ANOVA showed significant changes in the numer of writhing episodes of mice after the administration of the compound **3n** (*F*_4.38_ = 7.204, *p* < 0.001), **3p** (*F*_5.54_ = 7.257, *p* < 0.0001), and **3s** (*F*_.,49_ = 14.17, *p* < 0.0001). Post-hoc Tukey’s test confirmed a significant reduction in the writhing episodes of mice after the administration of the compound **3n**—0.1, 0.05, and 0.025 ED_50_ (*p* < 0.001, *p* < 0.01, *p* < 0.05), **3p**—0.1, 0.05 ED_50_ (*p* < 0.001), and 0.025, 0.0125 ED_50_ (*p* < 0.05) and **3s**—0.1, 0.05 ED_50_ (*p* < 0.001), and 0.025 ED_50_ (*p* < 0.01)

**Fig. 7:**
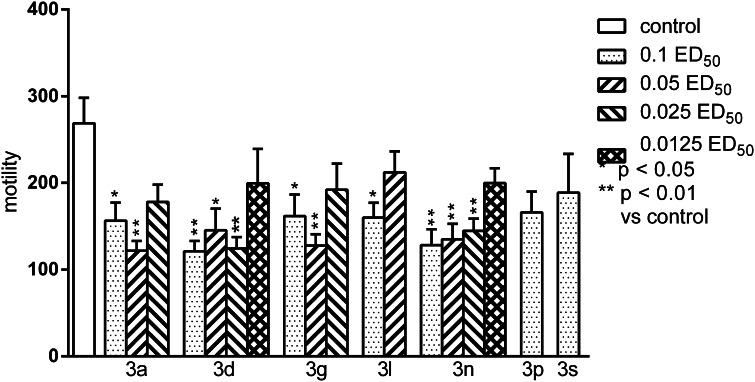
The influence of the tested compounds on the spontaneous locomotor activity of mice. The results are expressed as mean ± SEM of a group of 6–14 mice. One-way ANOVA showed significant changes in locomotor activity of mice after the administration of the compound **3a** (*F*_3,29_ = 5.999, *p* < 0.01), **3d** (*F*_4,35_ = 4.942, *p* < 0.01), **3g** (*F*_3,31_ = 5.6, *p* < 0.01), **3l** (*F*_2,25_ = 3.361, *p* = 0.051) and **3n** (*F*_4,37_ = 6.596, *p* < 0.001). Post-hoc Tukey’s test confirmed a significant reduction in motility of mice after the administration of the compound **3a** in the dose of 0.1 ED_50_ (*p* < 0.05) and 0.05 ED_50_ (*p* < 0.01), **3d**—0.1 ED_50_ (*p* < 0.01), 0.05, and 0.025 ED_50_ (appropriately *p* < 0.05, *p* < 0.01), **3g**—0.1 ED_50_ (*p* < 0.05) and 0.05 ED_50_ (*p* < 0.01), **3l**—0.1 ED_50_ (*p* < 0.05) and **3n—**0.1, 0.05, and 0.025 ED_50_ (*p* < 0.01)

All other tested compounds exerted significant antinociceptive activity in the writhing test (Fig. 6a, b). The effect was strong for all of the compounds and remained until the dose equivalent to 0.025 ED_50_. In the case of compound **3p,** a significant reduction in number of writhing episodes was also observed, when the compound was used at a lower dose of 0.0125 ED_50_. However, we observed significant impairment of motor coordination in the rota-rod test after dose of 0.1 ED_50_ of this compound, what can hinder the interpretation of this result as a significant analgesic effect. On the other hand, the administration of the compound **3p** did not cause any change in the spontaneous locomotor activity of the animals (Fig. 7), which would indicate that the compound **3p** disturbing coordination, does not change the motor activity. The antinociceptive activity of the tested compounds does not appear to be associated with endogenous opioid system because naloxone (5 mg/kg) nonselective opioid receptor antagonist did not alter the observed effects (data not presented).

Most of the tested compounds (with the exception of **3p** and **3s**) significantly decreased spontaneous motility of mice (Fig. 7). The noted effects of **3a** and **3g** were very strong and persisted up to 0.05 ED_50_, these of **3d** and **3n** up to 0.025 ED_50_ and compound **3l** decreased motility only at the dose of 0.1 ED_50_ (*p* < 0.05). None of the tested compounds inhibits amphetamine-induced hyperactivity (data not presented). It is necessary to underline that the tested compounds did not exhibit neurotoxicity because used in dose equivalent to 0.1 ED_50_ they did not disturb motor coordination of mice in the rota-rod test. The only exception was substance **3p**, discussed above. The lack of motor-impairing effects is important because it can change the results of other tests (e.g., motility tests) and affecting reliability of the tests results.

**Fig. 8:**
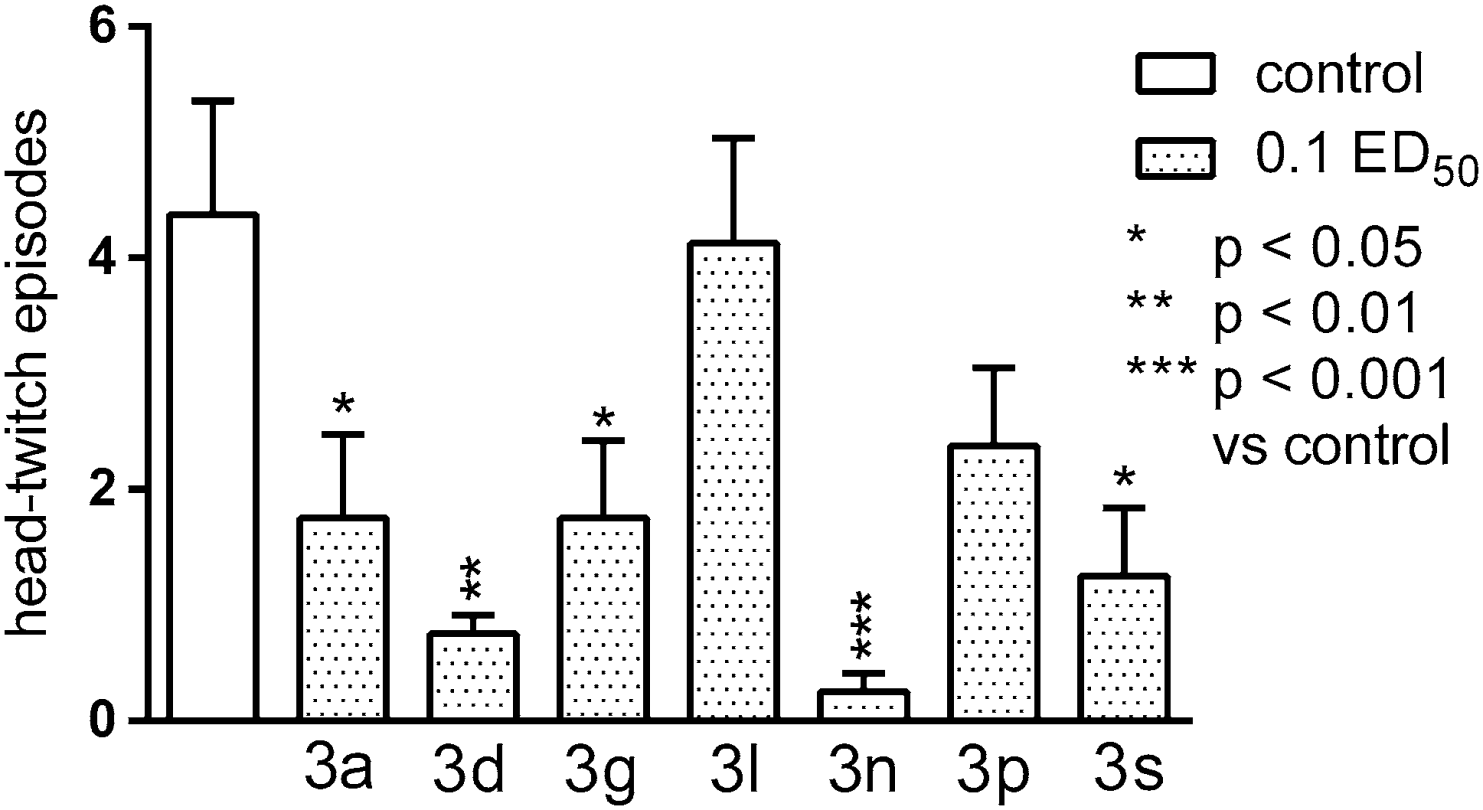
The influence of the tested compounds on the head-twitch responses evoked by L-5-HTP (230 mg/kg). The results are expressed as mean ± SEM of a group of eight mice. One-way ANOVA showed significant changes in the number of head-twitch episodes (*F*_7,56_ = 4.879, *p* < 0.001). The post-hoc Tukey’s test confirmed a significant decrease in the numer of head-twitch episodes after the administration of the following compounds in the dose of 0.1 ED_50_: **3n** (*p* < 0.001), **3d** (*p* < 0.01), and **3a**, **3g**, and **3s** (*p* < 0.05)

The tested compounds only slightly affected body temperature of mice: used in a dose equivalent to 0.1 ED_50_ significantly lowered it, but only in 30-min of observation, and used at twice less dose increased it (**3p** and **3s**) or have no effect (the others). Almost all tested compounds (except **3l** and **3p**) and to varying degrees (the strongest effect for **3n** compound, *p* < 0.001) suppressed L-5-HTP-induced head-twitch episodes (Fig. 8), suggesting some connections with serotonin system. The tested substances failed to protect against clonic seizures, tonic convulsions, and death in PTZ-induced model of seizures.

The results of the pharmacological investigation showed that both investigated series exerted significant influence on the central nervous system of laboratory animals. The most important seems to be their strong CNS depressive, antinociceptive, and serotonergic effects. The observed effects on the CNS of mice seem to be connected primarily with serotonergic neurotransmission, since almost all compounds (except **3l**, **3p**) inhibited significantly

L-5-HTP-induced head-twitches. The drug-elicited head-twitch response (HTR) (Corne *et al.*, 1963; Corne *and* Pickering, 1967) is a selective behavioral model for 5-HT2 agonist activity in rodents, and several previous studies have established that direct and indirect 5-HT agonists induce this effect (Colpaert and Janssen, 1983; Darmani *et al.*, 1990a, b, 1992; Fantegrossi *et al.*, 2004; Peroutka *et al.,* 1981). Furthermore, 5-HT2 receptor antagonists selectively block HTR (Fantegrossi *et al.,* 2004; Handley and Singh, 1986; Lucki *et al.*, 1984), and their potency is highly correlated with the antagonist’s affinity for 5-HT_2_ receptors (Ortmann *et al.*, 1982; Peroutka *et al.*, 1981). In addition, most of the tested compounds inhibited the motility of animals and changed body temperature of normothermic mice, which also may confirm the involvement of serotonin system.

### Structure–activity relationship

**Fig. 9:**
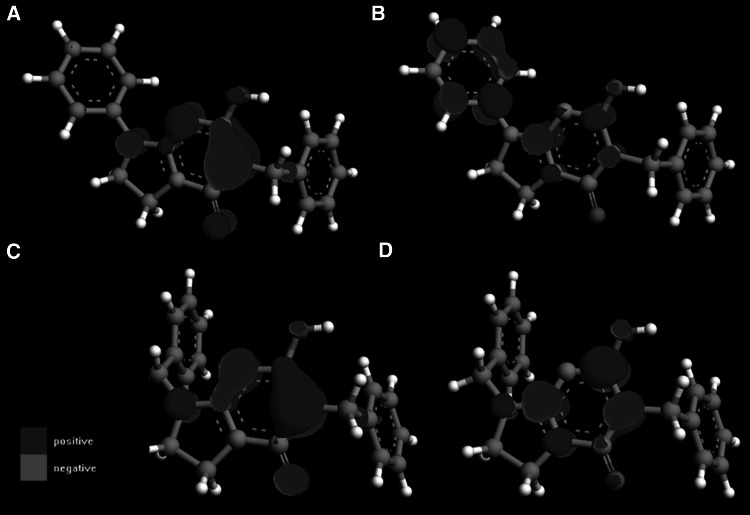
HOMO (**a**, **c**) and LUMO (**b**, **d**) orbitals for **3a** (**a**, **b**) and **3l** (**c**, **d**)

**Fig. 10:**
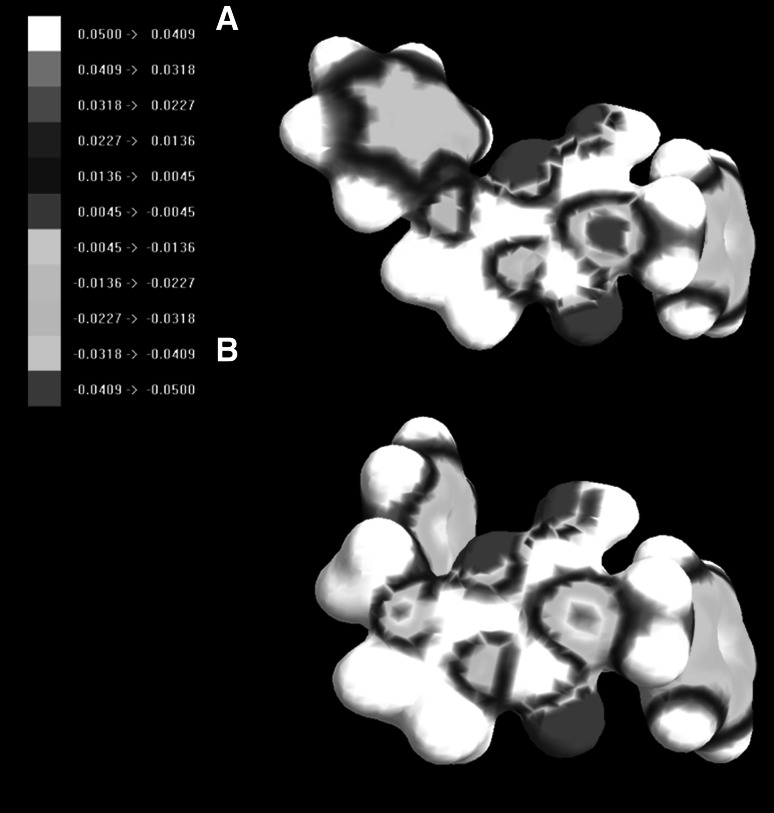
The map of the electrostatic potential (ESP) onto a surface of the electron density for **3a** (**a**) and **3l** (**b**)

The lack of activity of compound **3l** may be connected with the low blood–brain permeation. Furthermore, the presence of benzyl not phenyl substituent at the nitrogen N1 atom orients the pharmacophoric aromatic ring differently and it may constitute another explanation of the lack of acivity of **3l.** In order to further investigate the lack of activity of this componds, some structural and electronic parameters were calculated (Table 3). Compounds **3l** and **3x** have the greatest value of HOMO–LUMO gap. Furthermore, the map of HOMO and LUMO orbitals for the inactive compound **3l** is slightly different than for the acive compound **3a** (Fig. 9). The same concerns the distribution of electrostatic potential (Fig. 10). Next, compound **3l** belongs to the biggest compounds of the series and may be literally to expanded to fit to the binding pocket of the potential molecular targets. Values of polar surface area and polarizability cannot be connected with the lack of activity of **3l**.

**Table 3:** Parameters for structure–activity relationship studies

Compound	HOMO	LUMO	HOMO–LUMO gap	PSA	Molar volume	Polarizability
**3a**	−8.493	−0.064	8.429	56.14	245.2	36.70
**3b**	−8.652	−0.353	8.300	56.14	254.5	38.52
**3c**	−8.704	−0.352	8.352	56.14	254.5	38.52
**3d**	−8.696	−0.405	8.291	56.14	254.5	38.52
**3e**	−8.780	−0.599	8.180	56.14	263.80	40.35
**3f**	−8.646	−0.571	8.075	56.14	263.80	40.35
**3g**	−8.599	−0.102	8.496	56.14	260.40	38.45
**3h**	−8.566	−0.151	8.415	56.14	260.40	38.45
**3i**	−8.581	−0.067	8.514	56.14	275.60	40.21
**3j**	−8.480	−0.091	8.389	65.37	266.80	39.00
**3k**	−8.529	−0.128	8.400	65.37	266.80	39.00
**3l**	−8.552	0.110	8.662	52.98	261.20	38.53
**3m**	−8.628	−0.189	8.438	56.14	254.50	38.52
**3n**	−8.679	−0.368	8.311	56.14	263.80	40.35
**3o**	−8.731	−0.369	8.362	56.14	263.80	40.35
**3p**	−8.722	−0.421	8.301	56.14	263.80	40.35
**3q**	−8.806	−0.613	8.193	56.14	273.00	42.17
**3r**	−8.674	−0.582	8.093	56.14	273.00	42.17
**3** **s**	−8.626	−0.124	8.502	56.14	269.70	40.28
**3t**	−8.591	−0.172	8.419	56.14	269.70	40.28
**3u**	−8.608	−0.089	8.519	56.14	284.90	42.03
**3v**	−8.506	−0.108	8.398	65.37	276.10	40.83
**3w**	−8.553	−0.150	8.403	65.37	276.10	40.83
**3x**	−8.581	0.076	8.657	56.14	270.50	40.35

*HOMO* highest occupied molecular orbital, *LUMO* lowest unoccupied molecular orbital, *PSA* polar surface area

## Conclusions

Here, we present a series of antinociceptive compounds, designed as exerting their action through opioid receptors (non-classical opioid receptor ligands) but surprisingly devoid of opioid receptor activity. Searching of the molecular target to explain the antinociceptive properties will be the subject of our future studies. Further docking investigations are required to find their binding modes in potential targets and to determine, if they are orthosteric, allosteric, or dualsteric ligands. One main conclusion from the studies is that extension of the non-classical opioid receptor pharmacophore with the additional aromatic moiety results in the lack of opioid receptor activity. In addition to antinociceptive activity, most of the tested compounds were serotoninergic agents. The compounds exhibited favorable values of ADMET parameters for the activity in the central nervous system. The lack of central nervous system activity of compound **3l** may be attributed to its low blood–brain barrier permeation, unfavorable position pharmacophoric aromatic moiety, high value of HOMO–LUMO gap, and the overall size of the molecule.

## Electronic supplementary material

Below is the link to the electronic supplementary material.

Supporting information available with spectral data of the compounds. (DOCX 832 kb)
