# Supplementary material for: Synthesis, central nervous system activity, and structure–activity relationship of 1-aryl-6-benzyl-7-hydroxy-2,3-dihydroimidazo[1,2-a]pyrimidine-5(1H)-ones
Source: Med Chem Res. 2014 Mar 27;23(9):4221–37. doi: 10.1007/s00044-014-0993-1 (PMC4127001; doi:10.1007/s00044-014-0993-1)
Supplement: Supplementary file 1 — Supporting information available with spectral data of the compounds. (DOCX 832 kb) [file 44_2014_993_MOESM1_ESM.docx]

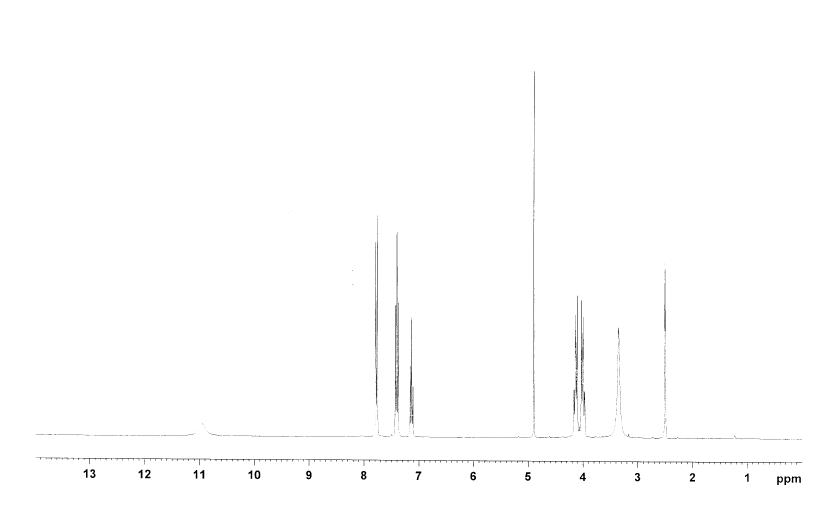


Fig. S1. ^1^H NMR spectrum of **3a.**


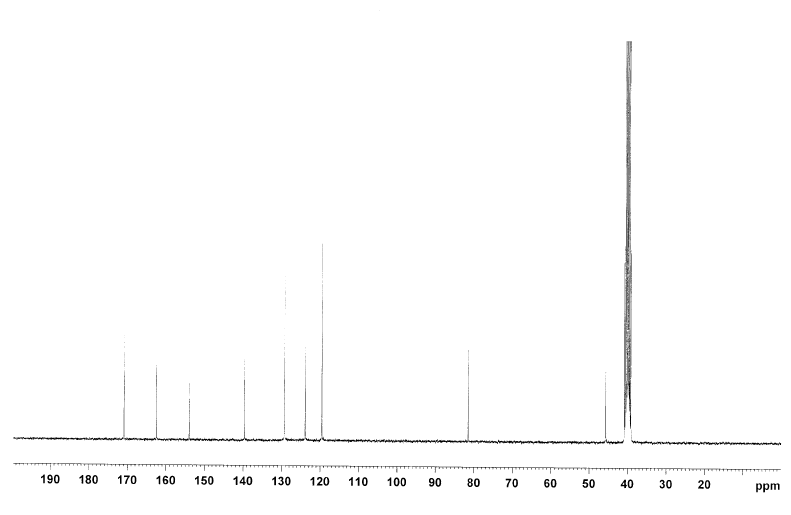


Fig. S2. ^13^C NMR spectrum of **3a**.


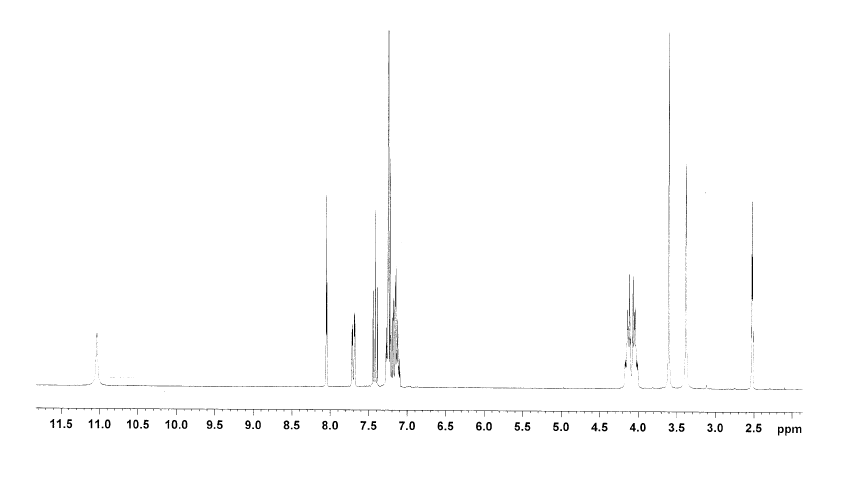


Fig. S3. ^1^H NMR spectrum of **3b.**


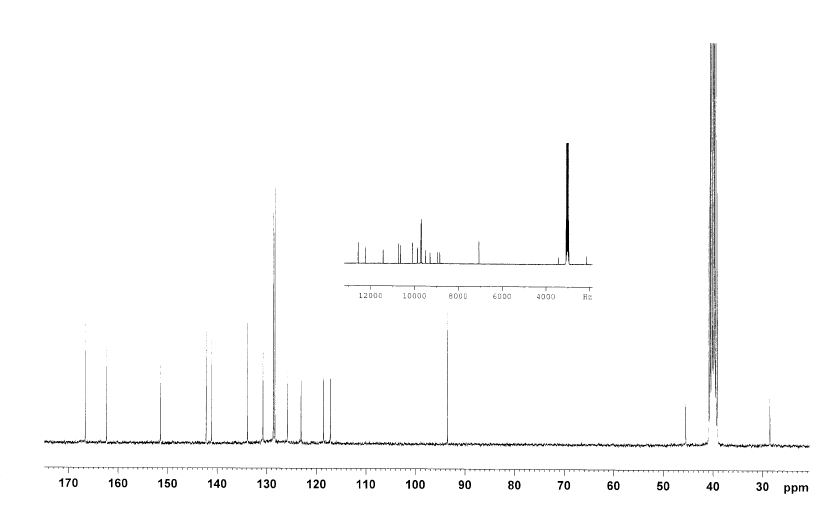


Fig. S4. ^13^C NMR spectrum of **3b**.


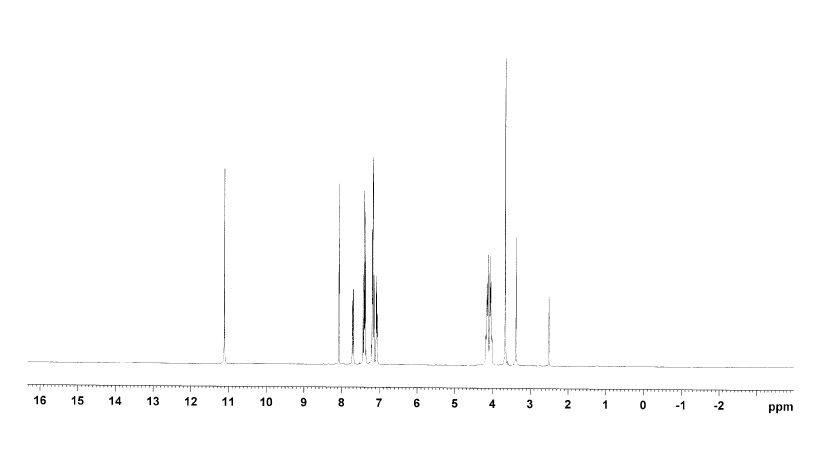


Fig. S5. ^1^H NMR spectrum of **3c.**


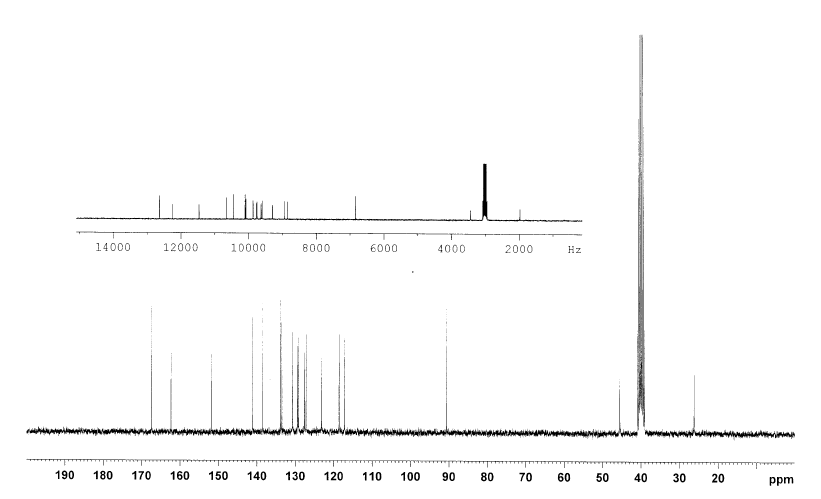


Fig. S6. ^13^C NMR spectrum of **3c**.


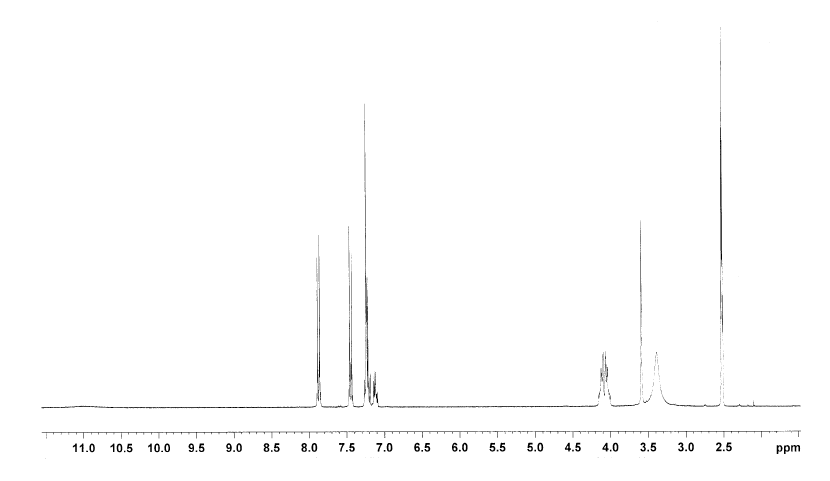


Fig. S7. ^1^H NMR spectrum of **3d.**


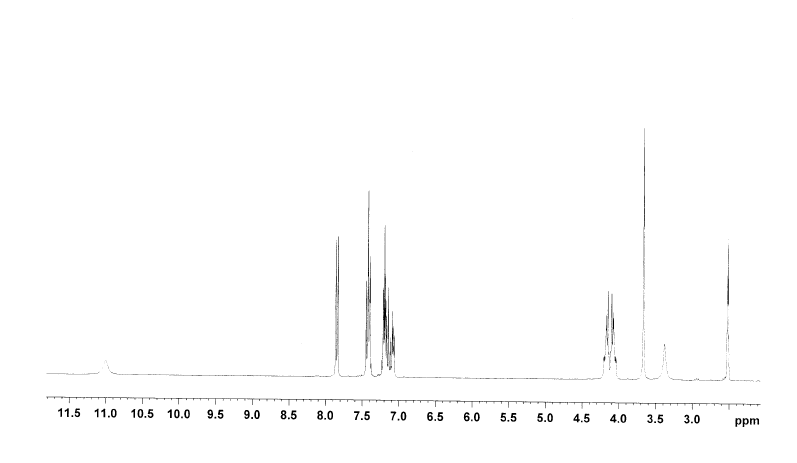


Fig. S8. ^1^H NMR spectrum of **3e.**


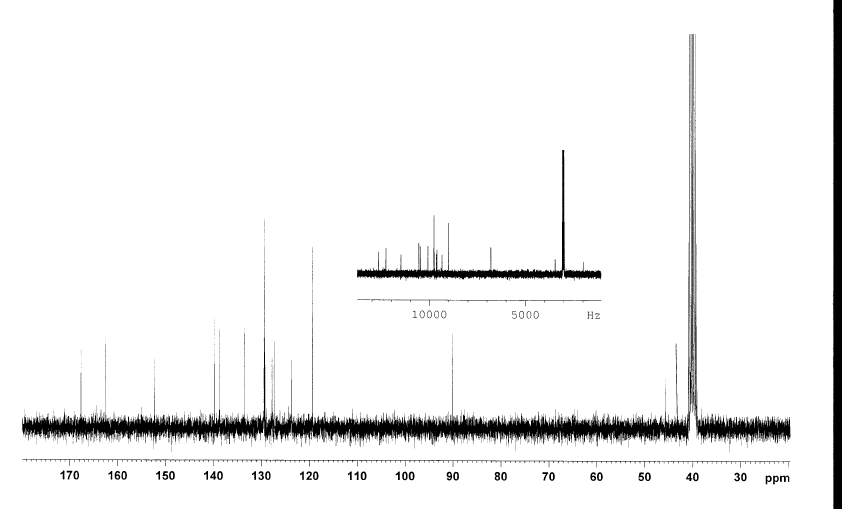


Fig. S9. ^13^C NMR spectrum of **3e**.


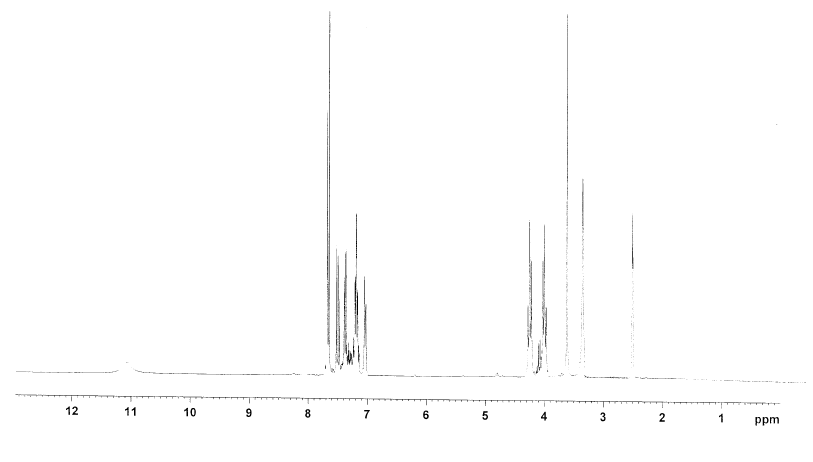


Fig. S10. ^1^H NMR spectrum of **3f.**


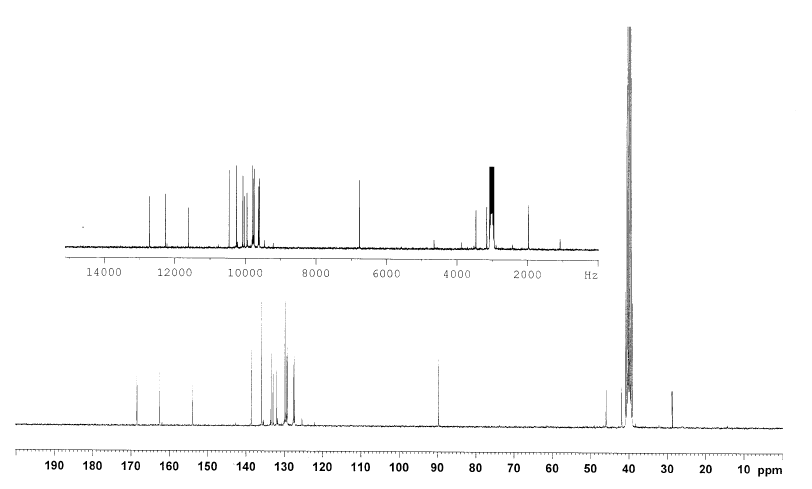


Fig. S11. ^13^C NMR spectrum of **3f**.


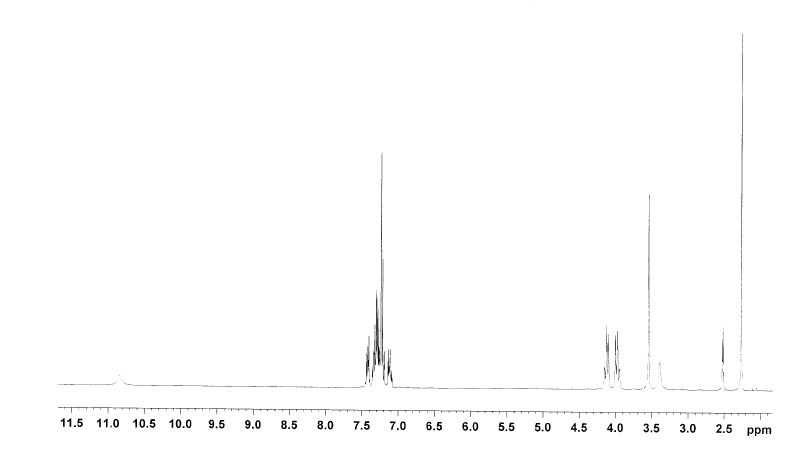


Fig. S12. ^1^H NMR spectrum of **3g.**


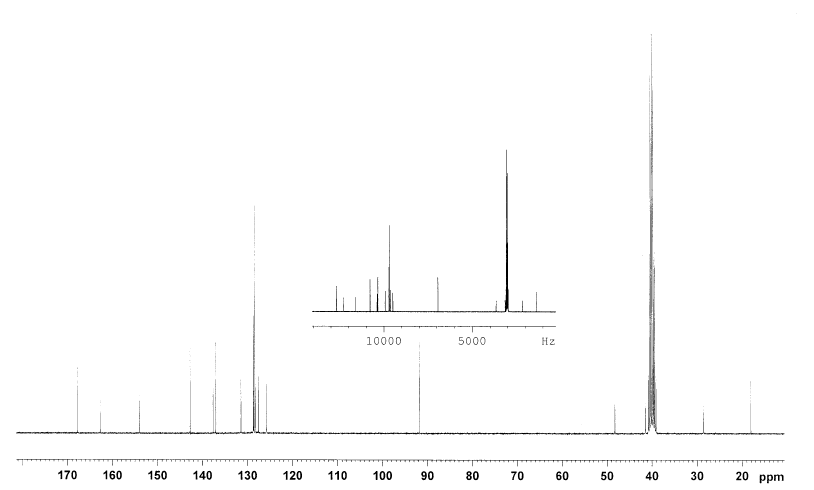


Fig. S13. ^13^C NMR spectrum of **3g**.


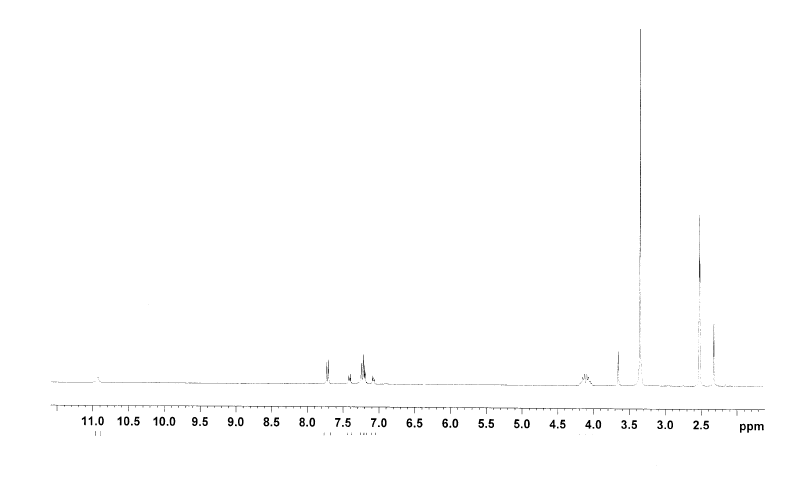


Fig. S14. ^1^H NMR spectrum of **3h.**


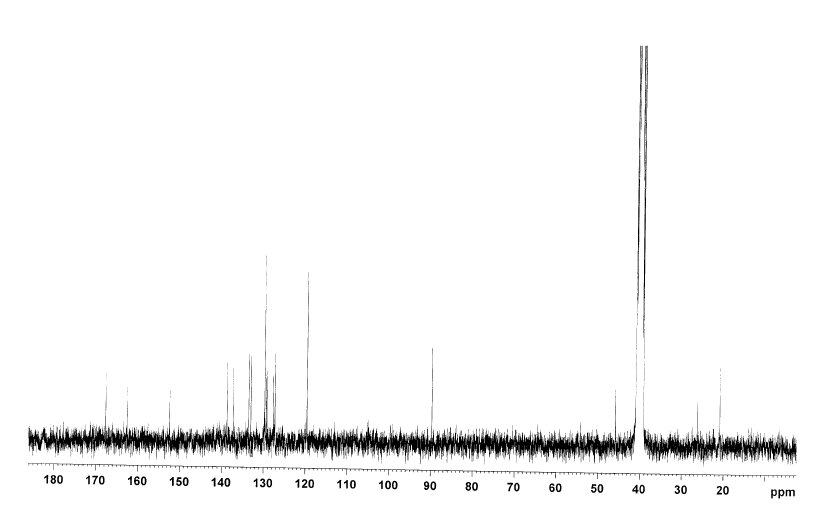


Fig. S15. ^13^C NMR spectrum of **3h**.


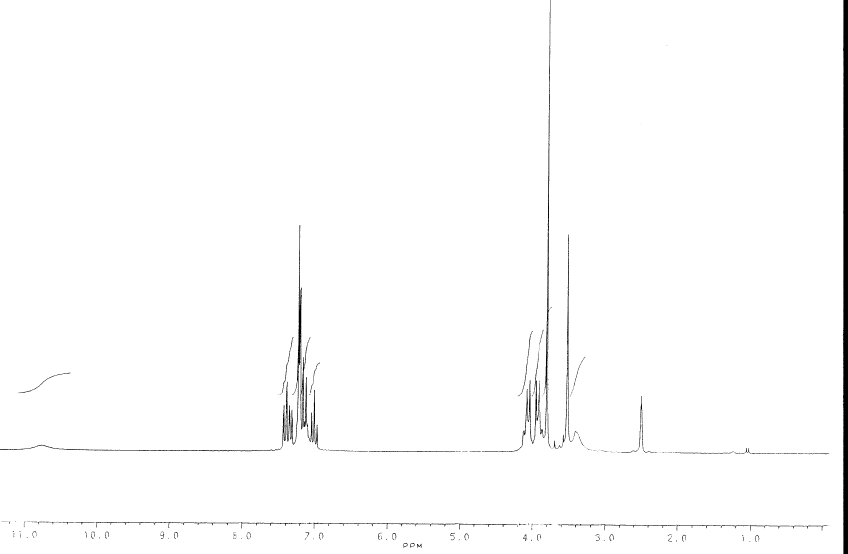


Fig. S16. ^1^H NMR spectrum of **3j.**


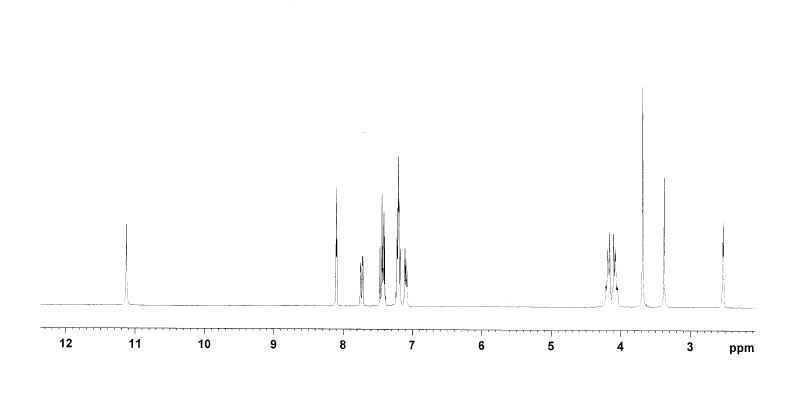


Fig. S17. ^1^H NMR spectrum of **3k.**


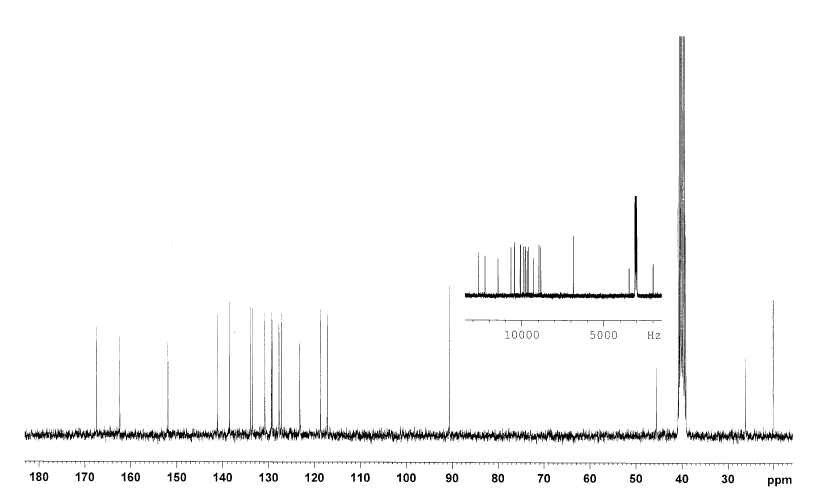


Fig. S18. ^13^C NMR spectrum of **3k**.


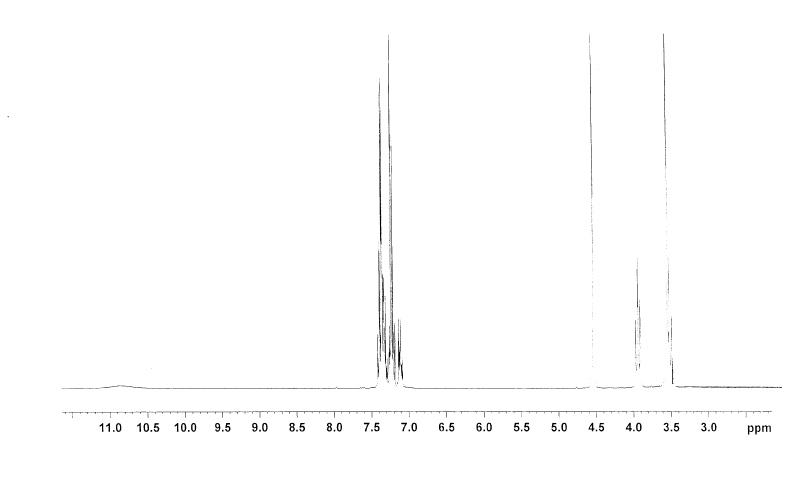


Fig. S19. ^1^H NMR spectrum of **3l.**


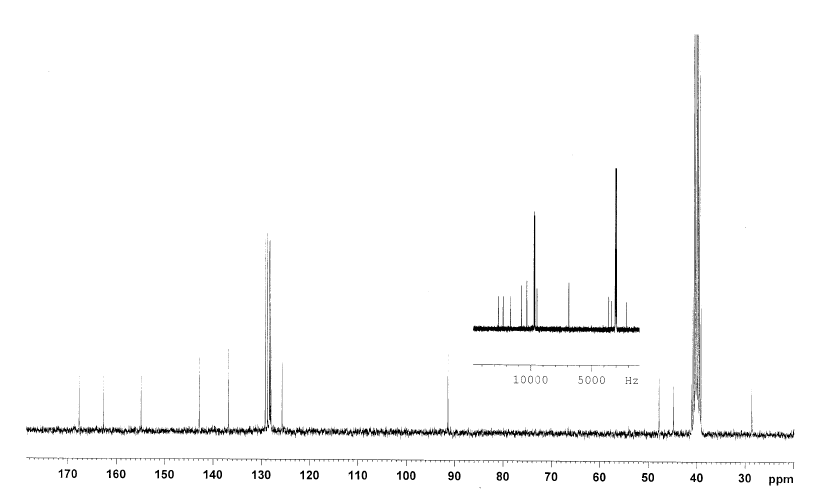


Fig. S20. ^13^C NMR spectrum of **3l**.


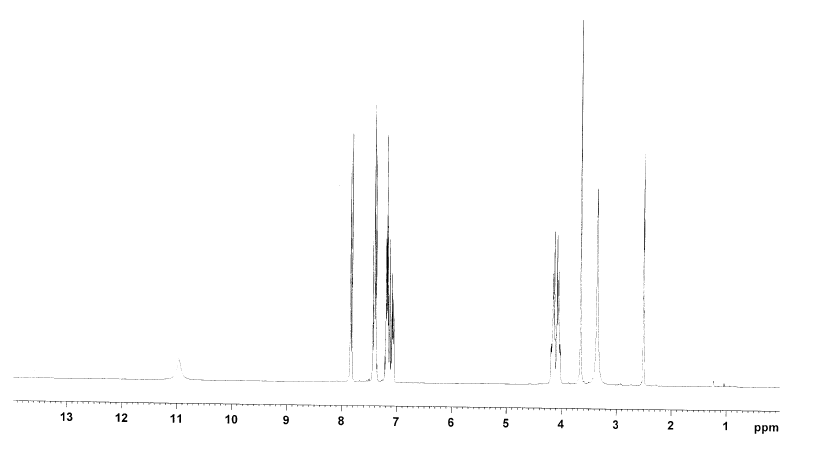


Fig. S21. ^1^H NMR spectrum of **3m.**

Fig. S22. ^13^C NMR spectrum of **3m.**


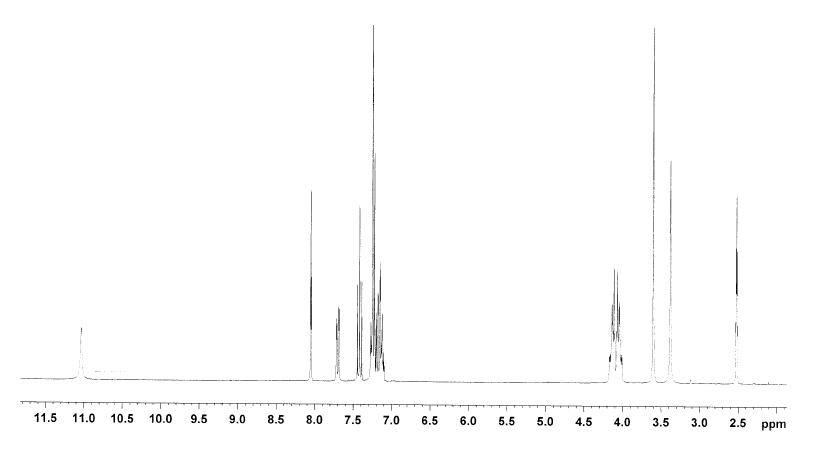


Fig. S23. ^1^H NMR spectrum of **3n.**


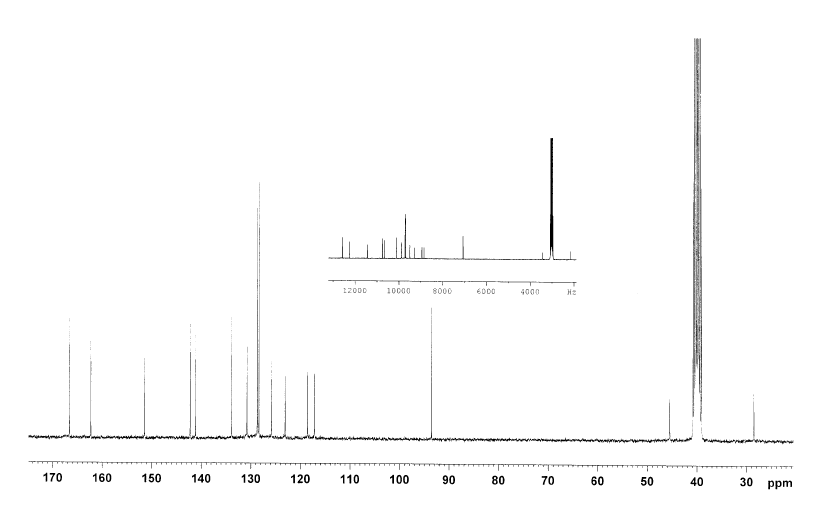


Fig. S24. ^13^C NMR spectrum of **3n.**


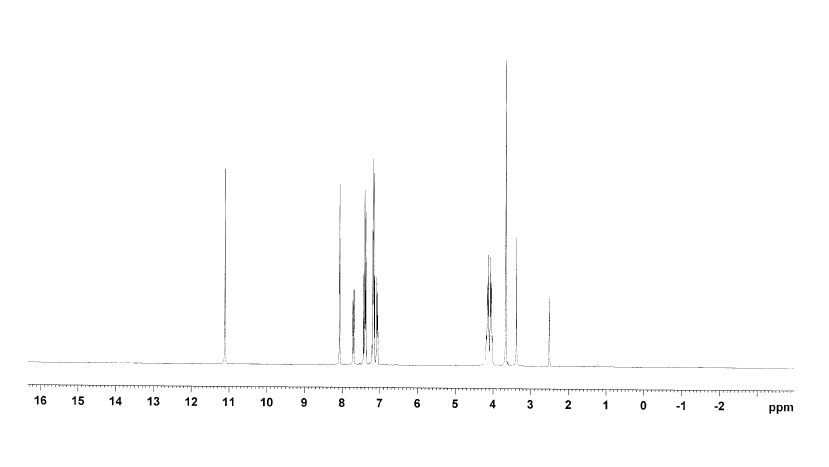


Fig. S25. ^1^H NMR spectrum of **3o.**


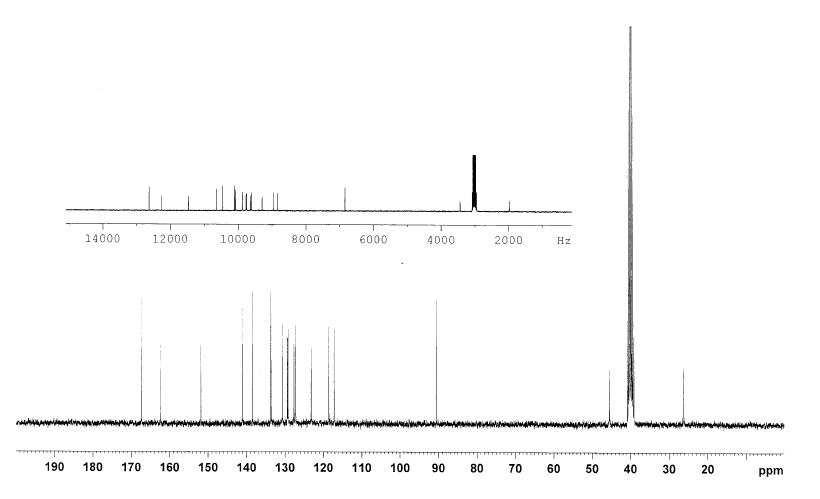


Fig. S26. ^13^C NMR spectrum of **3o.**


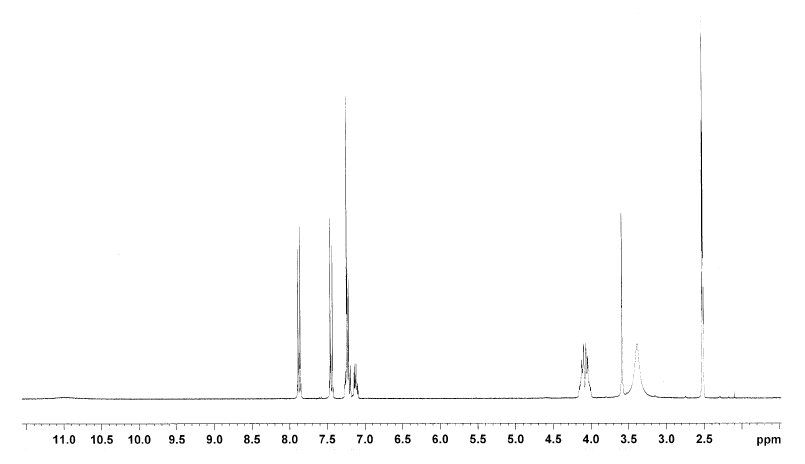


Fig. S27. ^1^H NMR spectrum of **3p.**


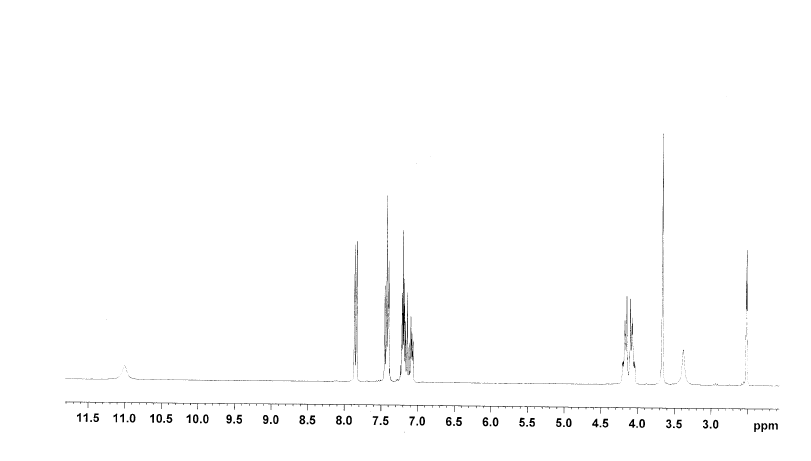


Fig. S28. ^1^H NMR spectrum of **3q.**


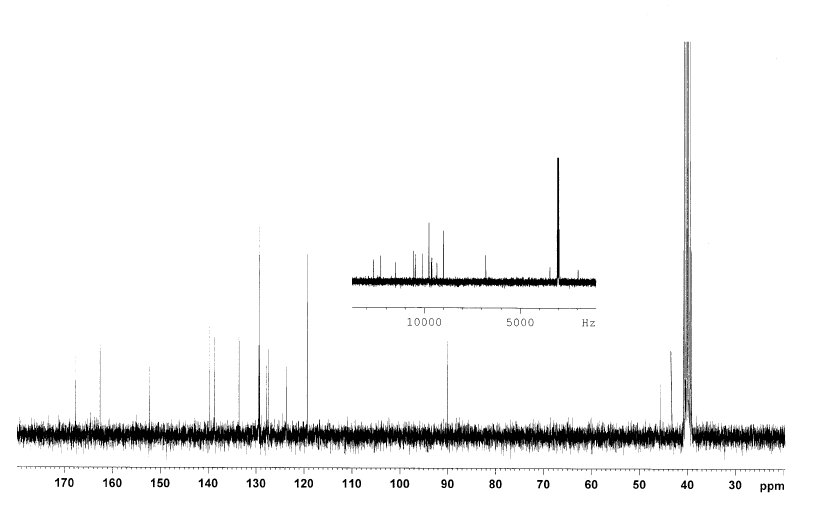


Fig. S29. ^13^C NMR spectrum of **3q.**


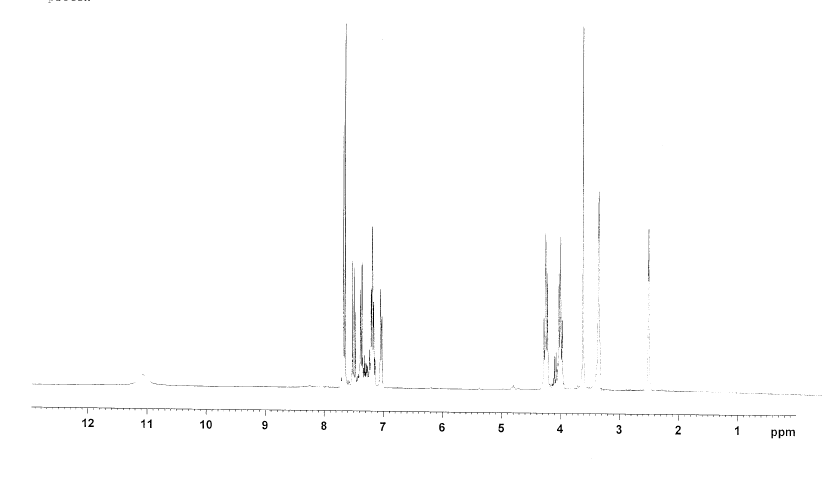


Fig. S30. ^1^H NMR spectrum of **3r.**


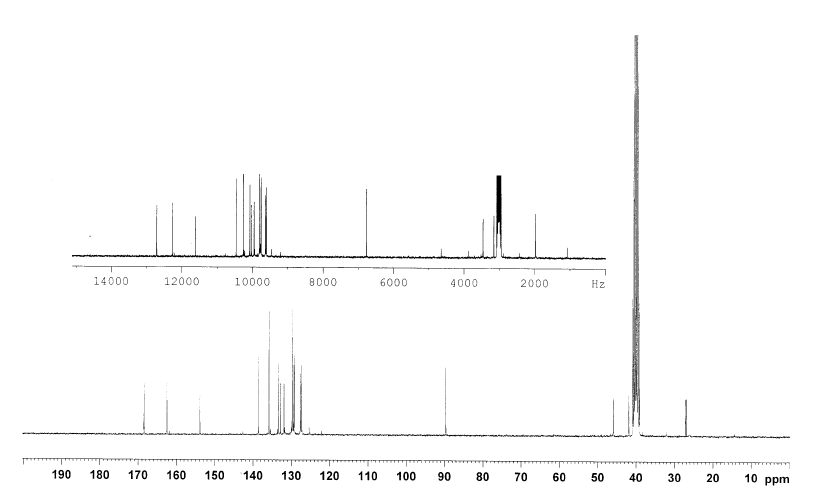


Fig. S31. ^13^C NMR spectrum of **3r.**


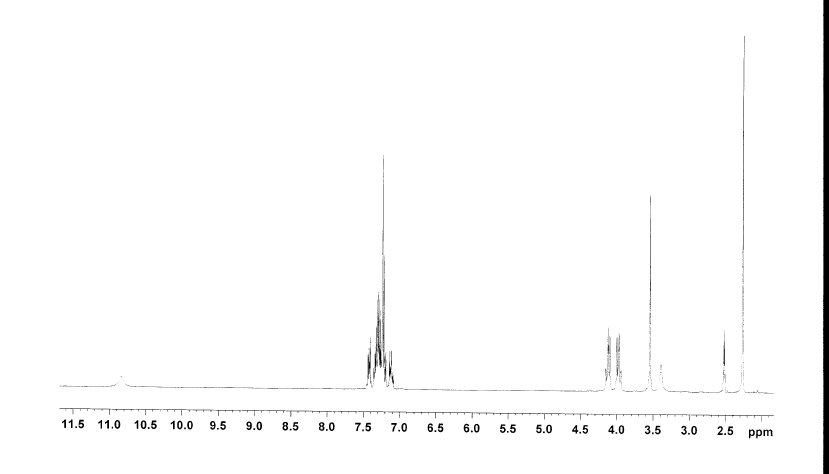


Fig. S32. ^1^H NMR spectrum of **3s.**


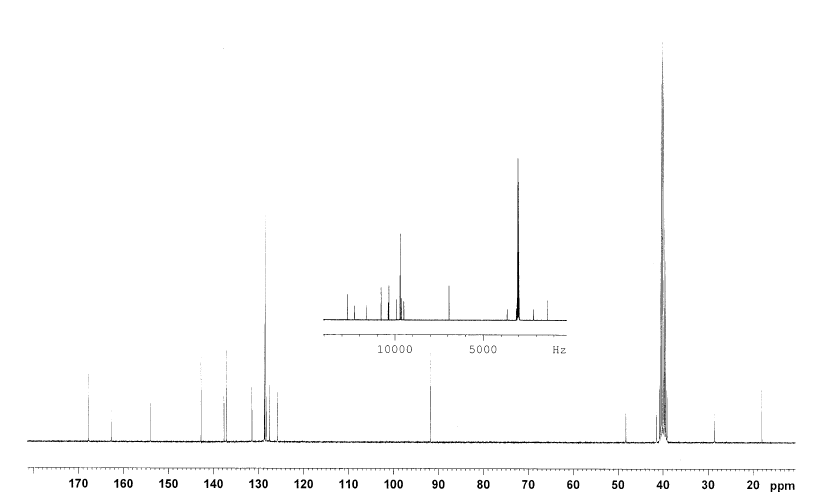


Fig. S33. ^13^C NMR spectrum of **3s.**


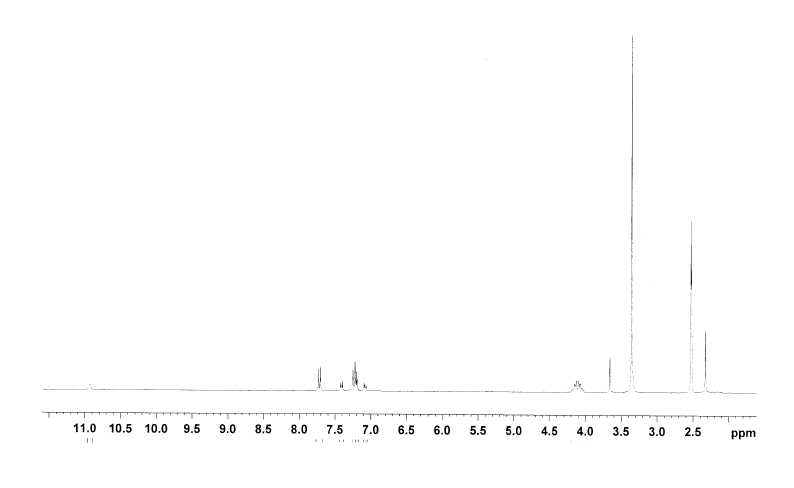


Fig. S34. ^1^H NMR spectrum of **3t.**


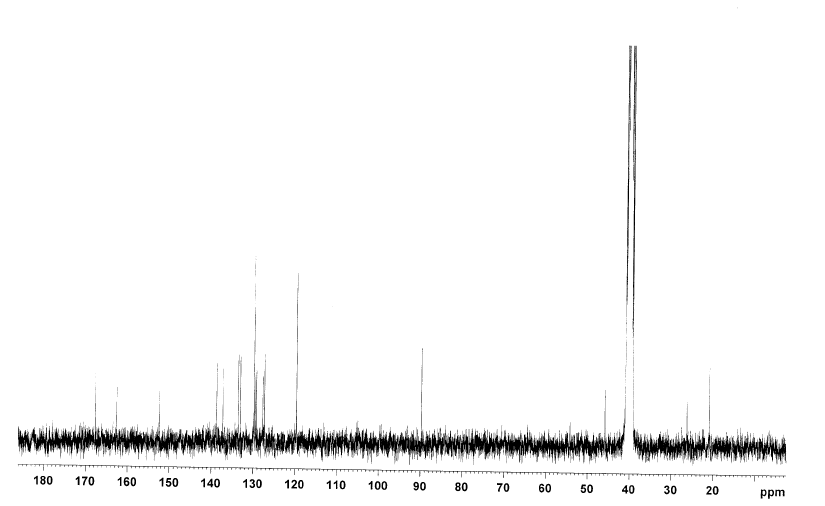


Fig. S35. ^13^C NMR spectrum of **3t.**


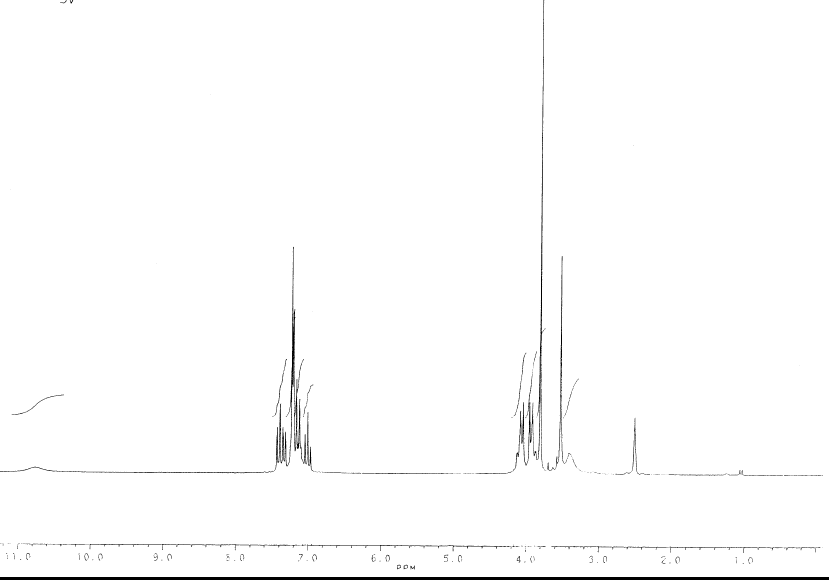


Fig. S36. ^1^H NMR spectrum of **3v.**


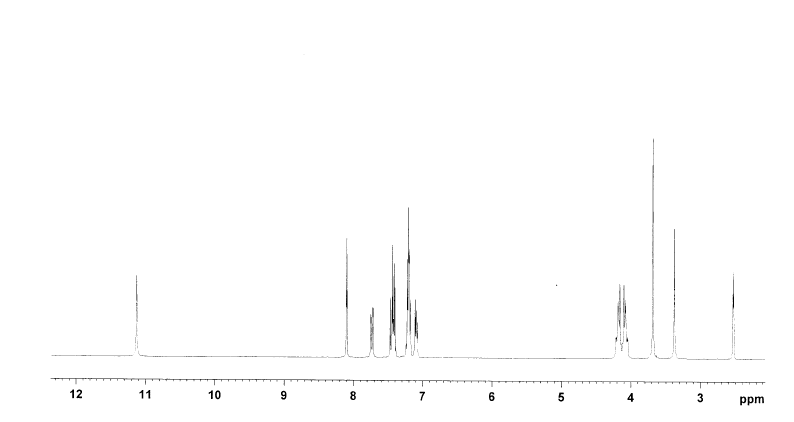


Fig. S37. ^1^H NMR spectrum of **3w.**


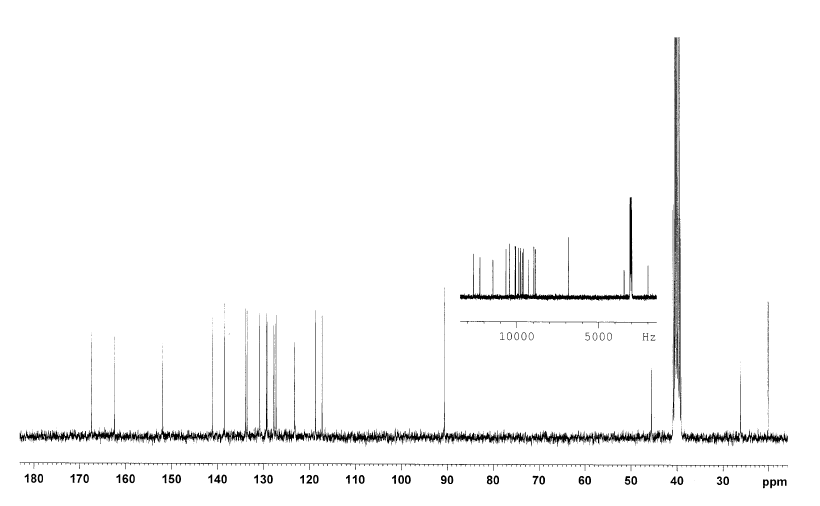


Fig. S38. ^13^C NMR spectrum of **3w.**


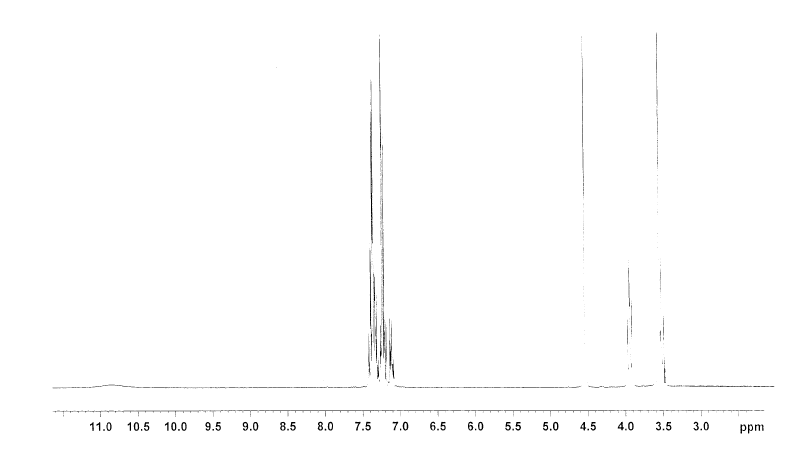


Fig. S39. ^1^H NMR spectrum of **3x.**


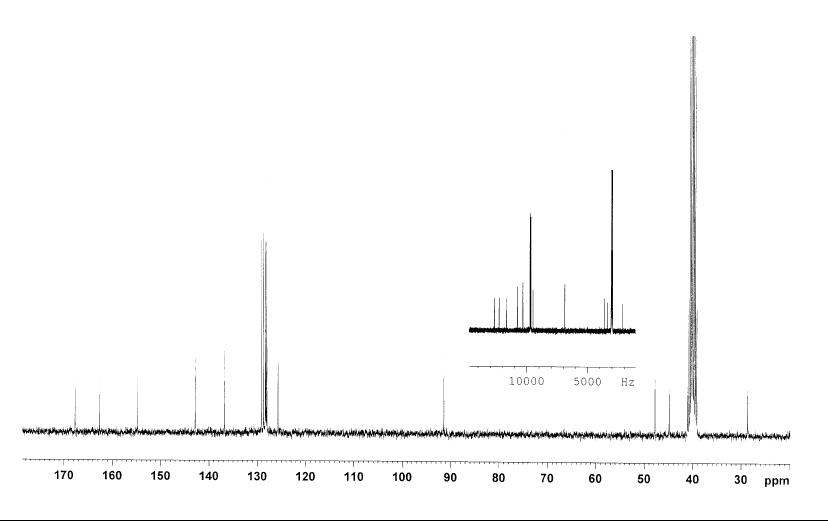


Fig. S40. ^13^C NMR spectrum of **3x.**
